# Novel Inhibitors Targeting Urease Promote Fungicidal and Antivirulence Effects in *Cryptococcus*

**DOI:** 10.1021/acsomega.6c03740

**Published:** 2026-07-01

**Authors:** Thayná Lopes Barreto, Nathália Evelyn Morais Costa, Nathalia Monteiro Lins Freire, Larissa Costa de Almeida, Rodrigo dos Anjos Miguel, Leticia Veras Costa-Lotufo, Gislaine da Silva-Rodrigues, Cleiton Moreira da Silva, Thiago Mendonça de Aquino, Eduardo Eliezer Alberto, Ângelo de Fátima, Kelly Ishida

**Affiliations:** a Department of Microbiology, Institute of Biomedical Sciences, University of São Paulo, 1374 Prof. Lineu Prestes Avenue, São Paulo, São Paulo 05508-000, Brazil; b Department of Chemistry, Institute of Exact Sciences, Federal University of Minas Gerais, 6627 Pres. Antônio Carlos Avenue, Belo Horizonte, Minas Gerais 31270-901, Brazil; c Research Group on Therapeutic Strategies - GPET, Institute of Chemistry and Biotechnology, Federal University of Alagoas, Lourival Melo Mota Avenue, Maceió, Alagoas 57072-900, Brazil; d Department of Pharmacology, Institute of Biomedical Sciences, 54544 University of São Paulo, 1524 Prof. Lineu Prestes Avenue, São Paulo, São Paulo 05508-000, Brazil; e Department of Organic Chemistry, Faculty of Science, Atatürk University, Erzurum 25030, Türkiye

## Abstract

Cryptococcosis is a life-threatening fungal disease caused by the *Cryptococcus neoformans* and *Cryptococcus gattii* species complexes, frequently associated with cryptococcal meningitis and high mortality. Urease is an important virulence factor that promotes intracellular survival and blood–brain barrier translocation, representing an antifungal target absent in humans. Here, a library of new synthetic urease inhibitors was evaluated against *Cryptococcus* growth and enzymatic activity. Four lead compounds AF19 (Schiff’s base), AF36 (benzoylselenourea), AF55, and AF57 (Biginelli’s adducts) displayed fungicidal activity and urease inhibition at micro- to nanomolar concentrations. Furthermore, using kinetics analysis and molecular dynamic simulations approaches revealed distinct inhibition modes: AF19 and AF57 acted as competitive nickel-coordinating inhibitors; AF36 exhibited noncompetitive inhibition via mobile-flap modulation; and AF55 behaved as a mixed-type inhibitor engaging both catalytic and allosteric sites. All leads reduced capsule thickness and increased membrane permeability, while AF55 and AF57 additionally suppressed melanin production. Together, the urease inhibitors AF55 and AF57 emerge as promising anticryptococcal candidates, combining a potent urease targeting, fungicidal activity, multivirulence inhibition, and favorable predicted pharmacokinetic and toxicological properties.

## Graphical abstract

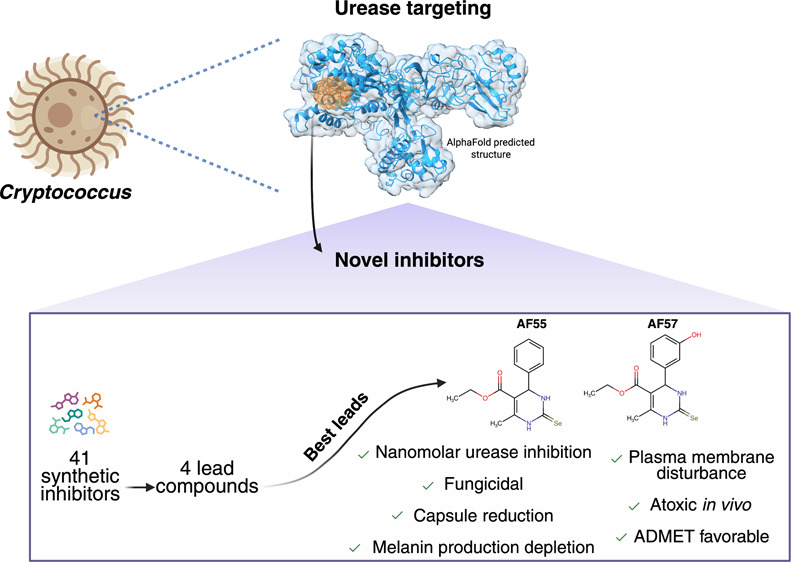

## Introduction

Cryptococcosis is a life-threatening invasive fungal infection caused by *Cryptococcus*, which is associated with high mortality and morbidity rates globally.^1^ The infection starts in the lungs, but its high tropism to the central nervous system (CNS) can lead to cryptococcal meningitis (CM), the most common and severe clinical manifestation.^1,2^ CM is an opportunistic infection caused by the *Cryptococcus neoformans* species complex, occurring in immunocompromised patients, especially those living with HIV/AIDS.^2^ However, an increasing number of cryptococcosis cases have been reported in immunocompetent individuals, mainly caused by the *Cryptococcus gattii* species complex.^3^

The current gold standard treatment protocol for CM recommends the combination of amphotericin B (AMB) and 5-fluorocytosine (5-FC) in the induction phase, while fluconazole (FLC) is indicated in the consolidation and maintenance phases. However, the therapy is associated with severe side effects.^2,4^ Increased resistance combined with the limited antifungal therapy for cryptococcosis underscores the urgent need to identify novel antifungal targets and develop promising antifungal candidates, although there are great efforts to develop new therapies.^5^

The pathogenesis of cryptococcosis is associated with virulence factors like the capsule, melanin, biofilms, and secretion of enzymes such as phospholipase, protease, nuclease, and urease.^6^ The urease (hydrolase, EC 3.5.1.5) catalyzes the hydrolysis of urea into ammonia and carbamate and has been described as a key virulence factor for brain invasion by *C. neoformans* yeasts by locally damaging the cells in the blood–brain barrier (BBB), weakening the endothelial tight junctions that improve the cryptococcal passage to CNS.^7,8^ Yeasts can also produce urease to survive inside macrophage phagolysosomes and be carried through the bloodstream and delivered directly to CNS by exocytosis (Trojan horse model).^9^ Additionally, urease production by *C. neoformans* in lungs increases accumulation of immature dendritic cells and promotes nonprotective inflammatory response.^10^

Urease is naturally absent in humans, minimizing the potential side effects of new drugs without interacting with human enzymes, thus representing an attractive antimicrobial target.^11^ Acetohydroxamic acid (AHA) remains the only approved drug used to treat urinary infection caused by urease-producing bacteria, such as *Proteus mirabilis*, and often, the therapy is associated with other antibacterial agents.^11^ Numerous studies have highlighted urease inhibition in pathogenic bacteria by natural products or synthetic compounds.^12–15^ However, most urease inhibitors have been tested against Jack bean urease (JBU, from *Canavalia ensiformis*), and despite the high sequence identity in the active site among different ureases, local folding variations may result in significant differences in the inhibition constant of ligands.^12^

Fungal urease inhibitor studies are still limited in the literature, but some compounds have demonstrated inhibitory effects on urease-producing fungi, especially *C. neoformans*.^16–19^ Our research group showed effective antifungal and antiureolytic activity of novel synthetic benzoylselenoureas (BSUs) against *C. neoformans*.^20^ Here, we explored the BSU AF36 and other novel synthetic urease inhibitors from a library with 34 synthetic compounds of different chemical classes. Then, after antifungal activity screening, four lead compounds (AF19, AF36, AF55, and AF57) were selected based on their higher inhibitory effect on fungal cell and urease inhibition at nanomolar level. These compounds presented different mechanisms of action and physiological effects on the *Cryptococcus* yeasts as the viability loss and inhibition of other virulence factors. Molecular docking and dynamic simulations and pharmacokinetic and toxicity profiles of the leads were predicted, highlighting AF55 and AF57 as potential candidates for anticryptococcal drug development.

## Results and Discussion

### Selenium-Containing Urease Inhibitors Present Higher Antifungal Activity on *Cryptococcus* spp. and Virulence Factor Inhibition

Antifungal activity screening of synthetic urease inhibitors (UREi) was performed against *C. neoformans* H99 strain using the RPMI and Christensen media for the determination of fungal growth and cell urease inhibition, respectively. From 56 UREi screened, only 17 inhibited the fungal growth at IC_90_ ≤150 μM, as molecules from Schiff’s bases,^21^ benzoylthioureas, benzoylselenoureas, and Biginelli’s adducts^22^ (Table S1). In the Christensen medium, UREi inhibited the cell urease with IC_URE_ of 2- to 4-fold lower than IC_90_ values for most of compounds tested, especially for the benzoylselenoureas^20^ and Biginelli’s adducts presenting 8- to 32-fold IC_URE_ lower than IC_90_ values (Table S1). Therefore, considering the best results with lower IC and IC_URE_ values and fungicidal effect (MFC ≤ 4 × IC_90_), four molecules (AF19, AF36, AF55, and AF57) were chosen as the lead compounds to explore antifungal effects and *Cryptococcus* urease inhibition (Table 1).

The four UREi were tested against clinical isolates of *C. neoformans* and *C. gattii* in RPMI and Christensen media with IC_90_ values ranging from 3 to 53 μM and IC_URE_ values ranging from 0.1 to 18 μM (Table 1 and Tables S2–S5), indicating the great potential of these molecules at inhibiting fungal growth and urease activity of *Cryptococcus* yeasts. All clinical isolates were susceptible to the standard antifungals 5-FC, AMB, and FLC (Tables S6 and S7), and the combinations of lead UREi with antifungals were considered indifferent (Tables S8 and S9).

AHA is the only approved drug used against *P. mirabilis* and *H. pylori* urease, and its effective inhibition to bacterial ureases is extensively described in the literature.^12,23,24^ AHA exhibited poor activity against *C. neoformans* growth and urease activity inhibition (IC_90_ and IC_URE_ ≥1705 μM) and lower inhibitory effect than the novel UREi tested (Table S1) and notably lower activity when compared with *H. pylori* (83 μM)^25^ and *Proteus mirabilis* (615 μM).^26^ Although with lower antifungal activity, AHA was included in all tests as a reference of standard urease inhibitor.

The inhibitory effect of the Schiff’s bases (Figure 1) was associated with the presence of hydroxyls in *R*_1_ and/or *R*_2_, i.e., the addition of hydroxyls was shown to increase antifungal activity (AF16 and AF18); however, a methoxy-group may have hampered the compound action (AF17) (Figure 1). Interestingly, the carbon chain elongation to 3 carbons and a hydroxyl group enhanced the activity in AF19, but H_3_C_2_N-substitution in *R*_1_ decreased activity in AF21 (Figure 1). Therefore, we selected AF19 as the lead compound from this class. Schiff’s base derivatives as a pyridine I are known to have anticryptococcal activity (Figure 1).^27^ Cinnamyl Schiff’s bases II and III (Figure 1) showed similar results against *C. neoformans* and *C. gattii* strains (MICs = 1.33–5.3 μg/mL),^28^ and their structural similarity to the allylamines (an antifungal class that targets the ergosterol biosynthesis) makes this class promising for antifungal therapy.^29^

Among benzoylselenoureas, we highlighted AF36 (no substitution, IC_90_ = 6.6 μM) and AF44 (3-position oxygen, IC_90_ = 3.3 μM), which showed the better antifungal activity. It is noteworthy that the elongation lateral chain at the para-position decreased the antifungal effect while 3-position substituted with chlorine, bromine, or oxygen, improved the activity (Table S1). However, considering the synthetic yield of the compounds AF36 and AF44 (75 and 25%, respectively),^20^ we have chosen AF36 as the lead compound for this class for the next experiments performed here.

**Table 1:**
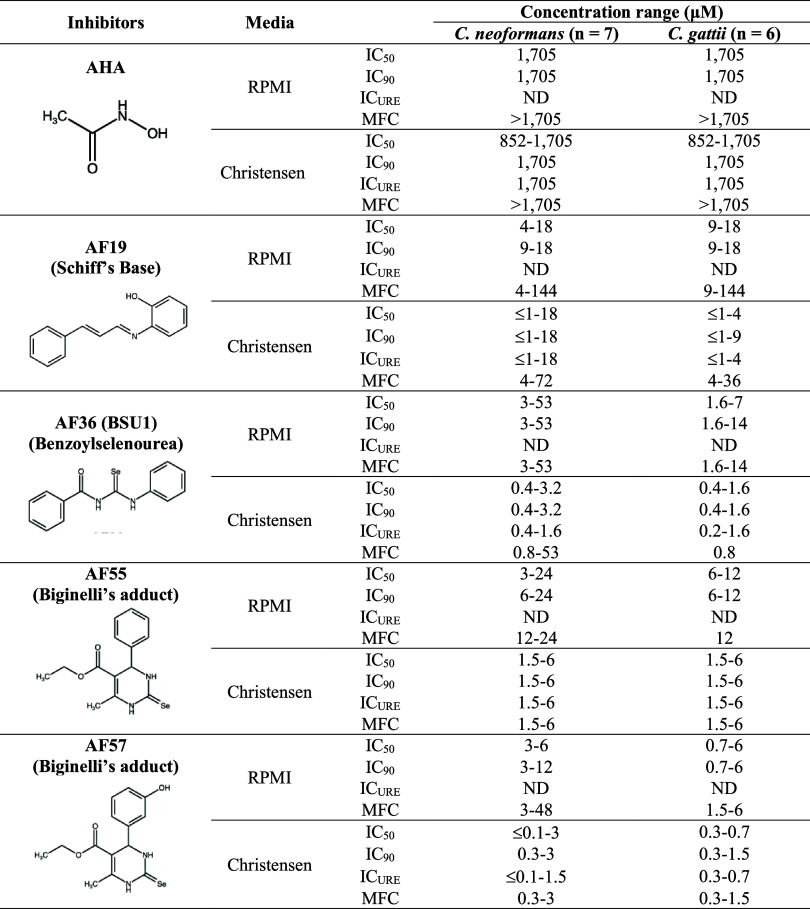
Susceptibility of *Cryptococcus neoformans* and *Cryptococcus gattii* Clinical Isolates to Urease Inhibitors in RPMI and Christensen Culture Media^a^

a ND: not determined; AHA: acetohydroxamic acid; IC_50_ and IC_90_: lowest concentrations that inhibit 50 and 90% of the fungal growth, respectively; IC_URE_: minimum inhibitory concentration on cell urease activity; MFC: minimum fungicidal concentration.

Selenium proved to be essential for the activity of Biginelli’s adducts on *Cryptococcus* yeasts, since its substitution by sulfur or oxygen did not show any activity (Table S1). Then, 4 selenium-Biginelli’s adducts showed an inhibitory effect at 6–48 μM (AF55–AF58). The presence or not of hydroxyl on 2-position did not change the activity (AF55 and AF56). The substitution in 3-position improved the activity (AF57) while 4-position decreased the inhibitory effect (AF58). These selenium compounds are analogs from dihydropyrimidinones (e.g., monastrol), an anticancer compound^30^ that has antifungal and antiureolytic potential described in the literature, but at higher concentrations (27–109 μM).^31,32^ Then, we selected AF57 due to lower IC values and higher synthetic yield (50%, data not shown); however, AF55 became also interesting due to similar antifungal and antiureolytic activity and structural potential of toxicity reduction.

**Figure 1:**
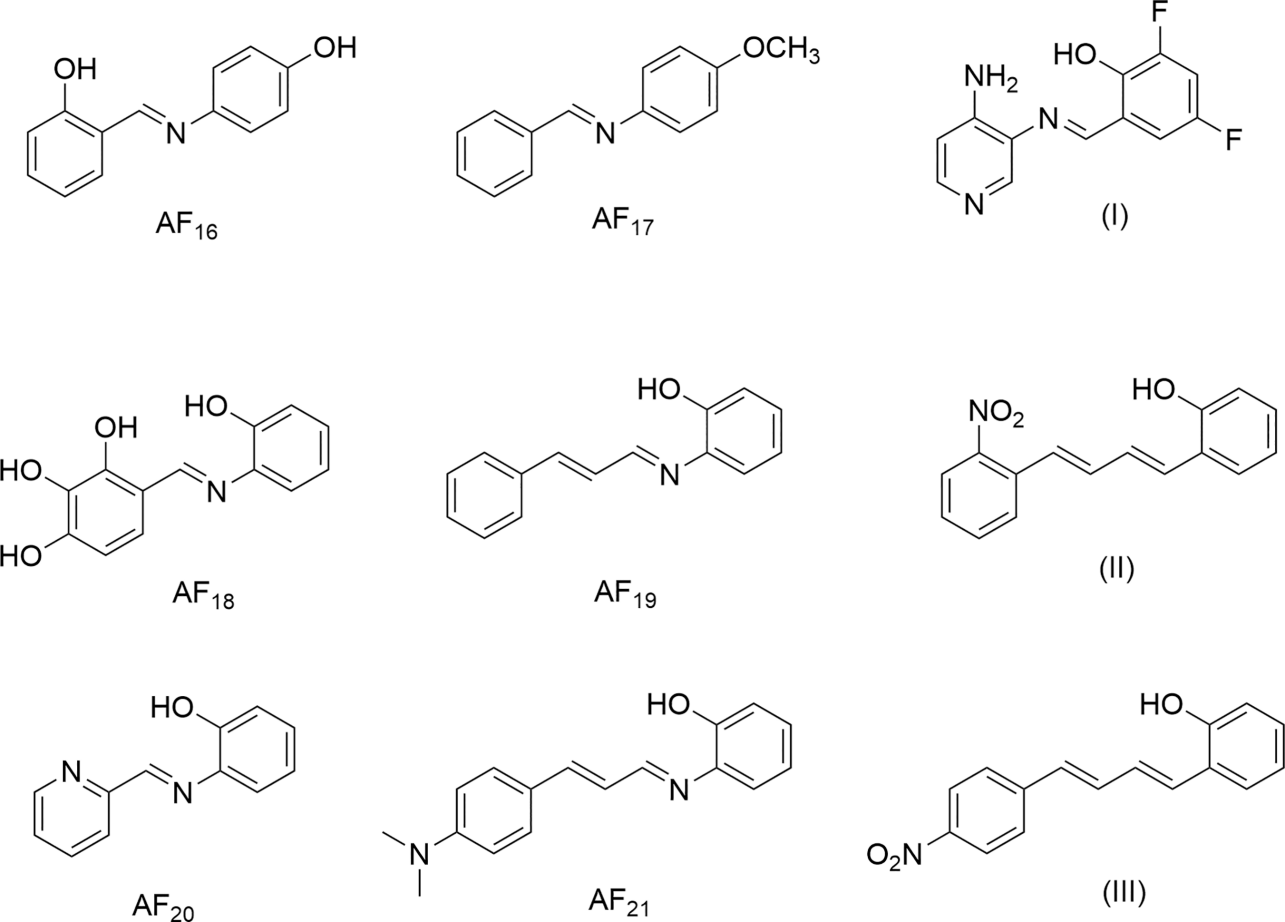
Schiff bases that demonstrated the most relevant structural aspects regarding activity against *C. neoformans* and *C. gattii* strains. Cinnamyl Schiff’s bases I, II, and II structure are also included for reference. For the other Schiff bases studied in the present study, see Table S1.

In addition to the inhibitory effect on growth and cell urease of *Cryptococcus*, we tested the UREi on other virulence factors such as biofilms, capsule, and melanin production. We highlighted the activity of the UREi AF36 on biofilm formation and mature biofilm of *C. neoformans* and *C. gattii* (Table 2). The UREi AF19 showed activity only on the biofilm formation of both species, but at higher concentrations, while AF55 and AF57 did not show activity on biofilm formation or on preformed biofilms. The dispersed biofilm cells were susceptible to all tested UREi, with DMIC values up to two dilutions higher when compared to planktonic cells (Table 2). On the other hand, the standard antifungal AMB presented better activity on the cryptococcal biofilm for both development phases (Table 2).

**Table 2:** Activity of Urease Inhibitors and Amphotericin B (AMB) on Planktonic Cells, Biofilm Formation, and on Sessile and Dispersed Cells from Mature Biofilm of *Cryptococcus* spp.^a^

	*C. neoformans* H99				*C. gattii* R265			
	formation		mature		formation		mature	
inhibitors	IC_90_	SMIC	SMIC	DMIC	IC_90_	SMIC	SMIC	DMIC
**AF19**	9	573	>573	9	9	573	>573	18
**AF36**	7	26	211	26	7	26	53	53
**AF55**	12	>397	>397	25	12	>397	>397	50
**AF57**	6	>378	>378	6	3	>378	>378	6
**AMB**	0.07	0.13	0.27	0.03	0.13	0.13	0.54	0.07

a IC_90_ and DMIC: lowest concentrations that inhibited 90% of fungal growth in planktonic and dispersed cells, respectively, obtained by the broth microdilution assay. SMIC: lowest concentrations that inhibited 50% of the metabolic activity of sessile biofilm cells by XTT reduction assay. The data are modal values obtained from biological triplicate and experimental triplicates and are expressed in μM.

Such as urease and biofilms, capsule and melanin production are important virulence factors for the establishment and spread of *Cryptococcus* infections. All lead urease inhibitors tested were able to significantly reduce the capsule thickness in *C. neoformans* and *C. gattii* (Figure 2A,B). However, only AF55 and AF57 totally inhibited melanization when yeasts were cultured on the minimal medium supplemented with L-DOPA (Figure 2C).

**Figure 2:**
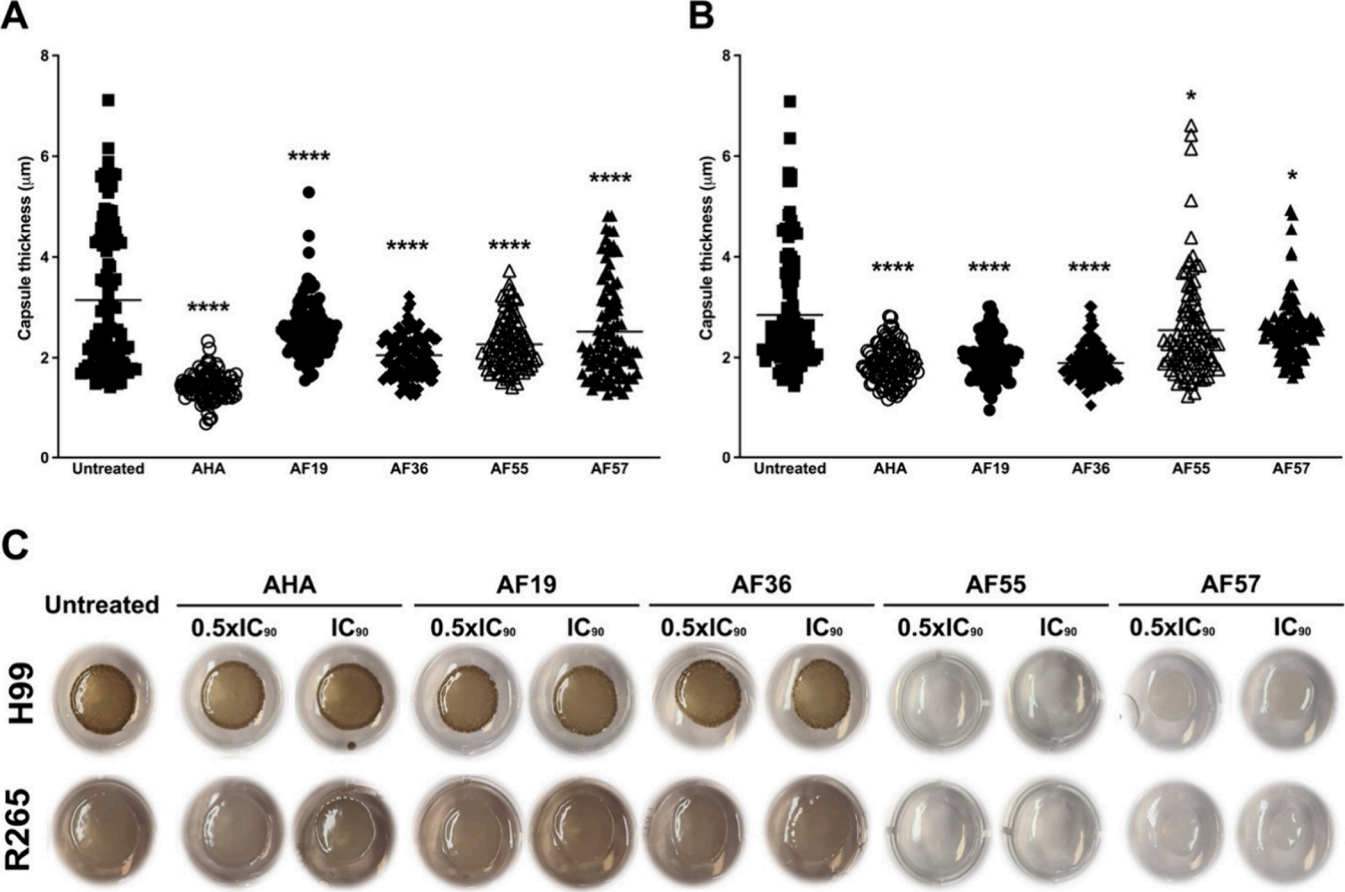
Capsule thickness and melanin production of *Cryptococcus neoformans* H99 and *Cryptococcus gattii* R265 after treatment with urease inhibitors. Capsule thickness of *C. neoformans* (A) and *C. gattii*­(B) treated with subinhibitory concentrations of urease inhibitors: AHA—acetohydroxamic acid (853 μM); AF19 (5 μM), AF36 and AF57 (3 μM), and AF55 (12 μM) in RPMI medium for 48 h at 35 °C. The capsule thickness was measured from 100 yeasts selected randomly from three independent experiments. *p < 0.05 and ****p < 0.0001 when compared with the untreated group (one-way ANOVA, Dunnet’s post-test). (C) Melanin production of yeasts untreated and treated with AHA (IC_90_ = 853 μM), AF19 (IC_90_ = 9 μM), AF36 (IC_90_ = 7 μM), AF55 (IC_90_ = 12 μM), and AF57 (IC_90_ = 6 μM) on the minimum medium supplemented with 1 mM L-DOPA, after 96 h of incubation at 35 °C.

Collectively, BSUs (especially AF36) and Biginelli’s adducts, both selenium-containing compounds, showed better inhibitory and fungicidal effects on *Cryptococcus* while also inhibiting yeast urease activity and other virulence factors. All UREi inhibited dispersion cells from biofilms and reduced capsule thickness, but only AF55 and AF57 additionally suppressed the melanin production.

Previous studies have shown that chalcogen compounds, particularly selenium-containing scaffolds, exhibit several biological properties, including antifungal activity.^33–35^ Ebselen and diphenyl diselenide are the most studied organoselenium presenting inhibitory activity against several fungal species at concentrations lower than 233 μM.^33^ Moreover, the organoselenium LQA_78 (ethyl 4-(benzylselanyl)-6-bromoquinoline-2-carboxylate) exhibited fungicidal activity, inducing necrosis/apoptosis and inhibiting several virulence factors, such as biofilm formation, capsule production, and melanin synthesis in *Cryptococcus*.^36^ The physiological effects observed for organoselenium compounds, including ebselen and LQA_78, may be associated with redox system imbalance and/or other mechanisms,^33,35,36^ inhibiting the thioredoxin reductase by covalent bond with enzyme catalytic cysteine.^37^ In addition to urease inhibition, the UREi compounds evaluated in the present study may also exert additional physiological effects in *Cryptococcus* spp. that contribute to their antifungal activity impairing fungal survival and invasion.

### Biginelli’s Adducts AF55 and AF57 Kill Quickly the *Cryptococcus* Yeasts and Cell Death Is Related to Increased Membrane Permeability

Concentrations of AF19 ≥IC_90_ were able to inhibit fungal growth close to the initial inoculum concentration, and a reduction in cell viability was observed to undetectable levels only at higher concentrations of 8x and 4 × IC_90_, after 60 h of exposure to *C. neoformans* and after 36 h to *C. gattii* (Figure 3A,E). This data indicates that in addition to inhibiting cell proliferation, the fungicidal action of AF19 was concentration- and time-dependent. AF36 has potent activity in reducing cell viability at concentrations ≥IC_90_ after 2 h of exposure on both strains (Figure 3B,F). The same activity time was required for the reduction of cell viability when treated with AF55 at 2 × IC_90_ (Figure 3C,G) while AF57 at concentrations ≥IC_90_ reduced the fungal viability of both species at undetectable levels after 12 h of exposure (Figure 3D,H). Additionally, all urease inhibitors at concentrations ≥IC_90_ were able to significantly increase the plasma membrane permeability comparable to AMB, a classical antifungal, and this phenomenon may be associated with the loss of yeast viability observed (Figure 3I,J).

**Figure 3:**
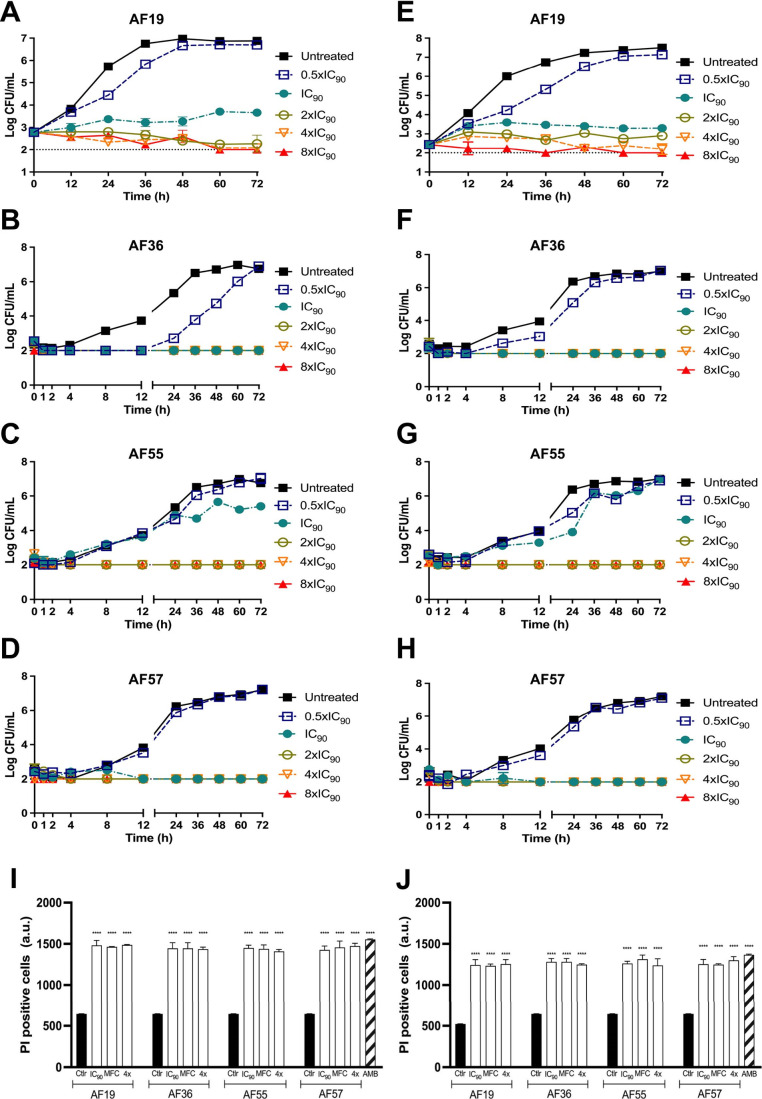
Viability of *Cryptococcus neoformans* and *Cryptococcus gattii* yeasts treated with UREi. Time-kill curves of *C. neoformans* H99 (A–D) and *C. gattii* R265 (E–H) treated with different concentrations of urease inhibitors AF19 (A,E), AF36 (B,F), AF55 (C,G), and AF57 (D,H) for 72 h at 35 °C. Detection limit is 2 log (dashed line). Plasma membrane permeability of *C. neoformans* (I) and *C. gattii* (J) treated with IC_90_ of AF19, AF36, AF55, and AF57 was analyzed using propidium iodide marker. Fungicidal concentration of amphotericin B (0.27 μM) was used as positive control. The assays were performed in technical duplicates and biological duplicates. ****p < 0.0001 when compared with the untreated group (one-way ANOVA, Dunnet’s post-test).

The development of fungicidal antifungal drugs is particularly important for the treatment of infections in immunocompromised patients. In these individuals, the immune system is often unable to effectively control fungal growth, making the host highly dependent on the drug’s ability to directly kill the pathogen. By rapidly reducing the fungal burden, fungicidal agents can improve clinical outcomes in severe infections. In addition, fungicidal drugs may reduce the likelihood of selecting resistant strains compared with fungistatic drugs, which only inhibit fungal growth, as can be observed in AMB and azoles.^1,39^ In fact, AMB is frequently recommended to treat the disseminated form, especially in immunocompromised patients, while FLC may be used to treat the pulmonary form of cryptococcosis.^4^

### Urease Inhibitors Suppress the *Cryptococcus neoformans* Urease Activity at Nanomolar Concentrations and the Inhibition Kinetics Reveal Different Enzymatic Inhibition Types

First, the steady-state kinetic analysis showed that *C. neoformans* urease follows a typical Michaelis–Menten saturation behavior, with calculated parameters of *K_m_* = 17.76 mM and *V*_max_ = 3.416 μmol ammonia/min/mg total protein (Figure S1). Next, enzymatic assays were performed using a range of inhibitor and urea concentrations to characterize the inhibition mechanism. The mechanism of action of the enzymatic inhibitors can be classified by the main types: competitive, noncompetitive, uncompetitive, and mixed inhibition.^45^ Then, we used the Lineweaver–Burk (LB) plot to determine the inhibition type of four lead urease inhibitors against *C. neoformans* urease. The inhibition kinetics has been observed in Figure S2, and all calculated *K_m_* and *V*_max_ values are described in Table S10.

The inhibitory effect of the standard UREi AHA and compounds (AF19, AF36, and all seleno-containing Biginelli’s adducts compounds—AF55, AF56, AF57, and AF58) was assessed on the urease obtained from the crude protein extract of *C. neoformans*. BSU derivatives (including AF36)^20^ and selenium-containing Biginelli’s adducts presented lower ureIC_50_ values (0.95–13.95 nM and 54.74–118.08 nM, respectively).

The benzoylselenourea AF36 presented to be a classic noncompetitive inhibition, in which the decreased *V*_max_ suggested an efficient reduction of the enzyme and possible binding to a different site, but it presented *K_m_* similar to control and lowest *K_i_* value between all UREi (*K_i_* = 32.32 nM, Table 3 and Figure S2B).

**Table 3:** Inhibition Kinetic Analysis of the Urease Inhibitors on Ureolytic Activity of *Cryptococcus neoformans* Urease from Cell Lysate^a^

inhibitors	ureIC_50_ ^b^(nM)	95% ureCI^b^ (nM)	inhibition type	*K_i_* (nM)
AHA	478.77 ± 15.72	334.77–684.85	noncompetitive	2,312
AF19	139.65 ± 5.42	93.56–208.44	competitive	66.88
AF36	4.88 ± 4.25^c^	2.90–8.21^c^	noncompetitive	32.32
AF55	54.92 ± 4.41	26.50–113.77	mixed	149.1
AF56	118.08 ± 3.72	73.11–191.90	ND	ND
AF57	89.96 ± 3.49	53.81–151.34	competitive	64.3
AF58	54.74 ± 3.87	31.39–95.99	ND	ND

a ND, not determined. Inhibitory concentration of 50% (ureIC_50_) with their respective confidence intervals (95% ureCI) of the standard urease inhibitor (acetohydroxamic acid—AHA) and urease inhibitors tested on urease obtained from the crude protein extract of *C. neoformans* yeasts. Inhibition type and inhibition constant (Ki) were obtained from inhibition kinetic analysis.

b The values represent the average and standard deviation of three biological replicates and three technical replicates.

c Costa et al.^20^

From Biginelli’s adducts, we highlighted the antiureolytic activity of AF55 and AF57 combined with better fungal growth inhibition (Tables 1 and 3). The dihydropyrimidinones, as monastrol and analogs, are known to have antiproliferative potential, mainly against cancer cells, and inhibitory activity on bacterial urease at concentrations of 11.76 μM for monastrol and 5.4 μM for analogs.^40^ The boronic Biginelli’s adducts containing a thiourea core showed JBU inhibition ranging from 132 to 168 μM,^41^ and our results with selenomonastrol analogs presented higher activity on *C. neoformans* yeast with a potent urease inhibition by AF55 and AF57. Although the UREi AF55 and AF57 belong to the same chemical class (selenium-containg Biginelli’s adducts), they presented different enzymatic inhibition types, highlighting the particularity of each molecule. AF55 exhibited a mixed type of inhibition, with a reduction on *V*_max_ and consequently reduction of enzyme efficiency and increased *K_m_* and *K_i_* value of 149.1 nM (Table 3 and Figure S2C). AF57 presented a competitive inhibition profile with constant *V*_max_, increased *K_m_*, and *K_i_* of 64.3 nM, comparable to values observed for AF19 (Table 3 and Figure S2D).

Among UREi, AF19 exhibited the lowest enzyme inhibition activity (Table 3), correlating with literature findings for JBU with new series of Schiff’s base derivatives (102–989 nM).^42^ AF19 showed a competitive inhibition profile, with no significant changes in *V*_max_, suggesting that its inhibitory effect can be overcome by the increase of substrate concentration, and increased *K_m_*, by reducing the affinity to the substrate presenting a *K_i_* value of 66.88 nM (Table 3 and Figure S2A).

In addition, the standard urease inhibitor AHA showed activity on the urease enzyme from *C. neoformans* with ureIC_50_ of 478.77 nM, and its inhibition was much lower (4–400 times) when compared to all novel UREi tested in this work (Table 3) and by Costa et al.^20^ This compound presented a noncompetitive inhibition profile with high *K_i_* value (2,312 nM), indicating the lower affinity to the *C. neoformans* urease when compared to all novel UREi tested (Figure S2E and Table 3). AHA is a structural analog of the substrate urea, and it is considered an irreversible inhibitor against bacterial urease. It is important to highlight that AHA inhibited urease activity of *Helicobacter pylori* (27.4 ± 1.2 μM),^43^ Jack Bean (31.7 ± 5.8 μM),^44^ *Proteus mirabilis* (120 μM)^26^ at higher concentrations compared with activity on *C. neoformans* urease.

Ebselen is another selenium-containing multifunctional drug that inhibits ureolytic activity of *H. pylori* as well as its derivatives.^46^ Besides, it inhibits *H. pylori* and Jack bean ureases at 1.5 and 0.4 μM, respectively, by covalently modifying the Cys rather than the His residue, the latter of which is known to be the active site of urease.^47^ In addition, ebselen derivatives presented high inhibitory activity on *H. pylori* urease (XBP2, IC_50_ = 140 nM) with significant gastric mucosal protective effects in murine model.^48^ Then, these studies suggested that compounds, specifically those selenium-containing scaffolds, may inhibit urease by forming a covalent S–Se adduct with a critical conserved Cys residue at the enzyme’s active site at the flap mobile. This mechanism is different from traditional nickel-chelating inhibitors, offering a novel approach to enzyme inhibition.^47^ Therefore, molecular docking and molecular dynamics analyses were used to propose a hypothesis for the interaction mechanism of the urease inhibitors studied here.

### Inhibition Mechanisms by Molecular Docking and Dynamic Simulations of Urease Inhibitors Using the AlphaFold Prediction Structure of *Cryptococcus neoformans* Urease

On the basis of the biological screening results, four lead compounds (AF19, AF36, AF55, and AF57) were selected for molecular modeling studies against *C. neoformans* urease. To explore their binding modes and interaction profiles at the urease active site, a homology model of *C. neoformans* urease was generated by using AlphaFold and refined, followed by molecular docking to predict the most favorable binding poses. The lowest-energy conformations for each compound were then subjected to molecular dynamics (MD) simulations to evaluate the complex stability and interaction persistence over time. This integrative approach was chosen to provide structural and energetic insights into the inhibitory mechanisms, guiding the rational design of the next-generation urease inhibitors.

For AF19 derivative, the MD simulations revealed that inhibition occurs through a direct coordination of its hydroxyl group to the Ni center via the oxygen atom, persisting for the entire simulation time (Figure 4A). This coordination was complemented by stable ionic interactions with His400 and His402, also maintained for the full duration, along with sustained hydrophobic contacts with Tyr537 observed during 90% of the simulation period (Figure 4B). The RMSD profile showed an initial adaptation phase within the first 40 ns, followed by stabilization, indicating that Ni-ligand coordination provided enhanced structural rigidity (Figure 4C). RMSF analysis demonstrated higher fluctuations in loop regions, while His400, His402, and Tyr537 remained notably stable, suggesting that ligand binding did not destabilize the catalytic core (Figure 4D). These observations reinforce the structural compatibility of AF19 within the active site and highlight the key role of Ni coordination and surrounding residues in maintaining complex stability.

**Figure 4:**
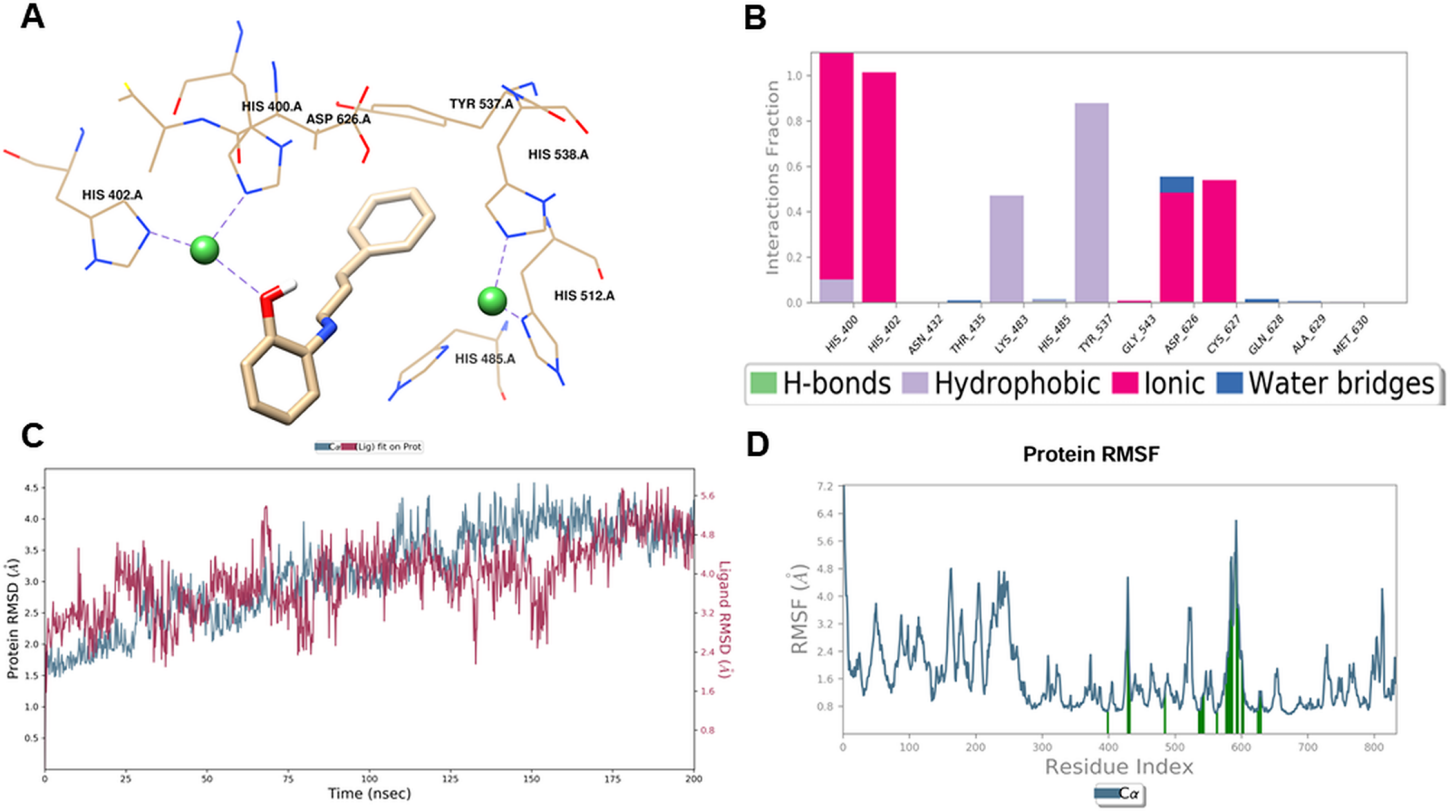
Three-dimensional representation of AF19 (A) bound to *C. neoformans* urease. Ligands are depicted in stick form, while the two Ni ions are highlighted as green spheres. Coordination to the metals is indicated by dashed lines. (B) The interaction profile of AF19 with the enzyme was monitored over the entire MD simulation. An interaction value of 1.0 denotes that the contact was maintained for the full simulation time, whereas values above 1.0 occur when the same residue establishes multiple contacts of the same type with the ligand. RMSD plots of the urease backbone and AF19 (C) in the catalytic cavity are shown, along with RMSF plots (D) of the urease–AF19 complex, where residues directly interacting with the ligand are marked by green vertical bars.

For the AF36 compound, the inhibition mechanism involved hydrophobic contacts with Met630 for half of the simulation time, supporting its affinity for the urease allosteric site (Figure 5A). Because of the spatial proximity between the allosteric and catalytic pockets, AF36 also achieved a persistent coordination of its carbonyl group to the Ni center via the oxygen atom for the entire simulation period (Figure 5B). RMSF comparison between the native enzyme (Figure 5C) and the AF36 (Figure 5D) complex revealed pronounced fluctuations between Met581 and His600 residues, corresponding to the mobile flap that controls access to the active site. These results suggest that AF36 binding induces conformational adjustments in this flap, potentially modulating substrate entry and, thereby, regulating ureolytic activity.

**Figure 5:**
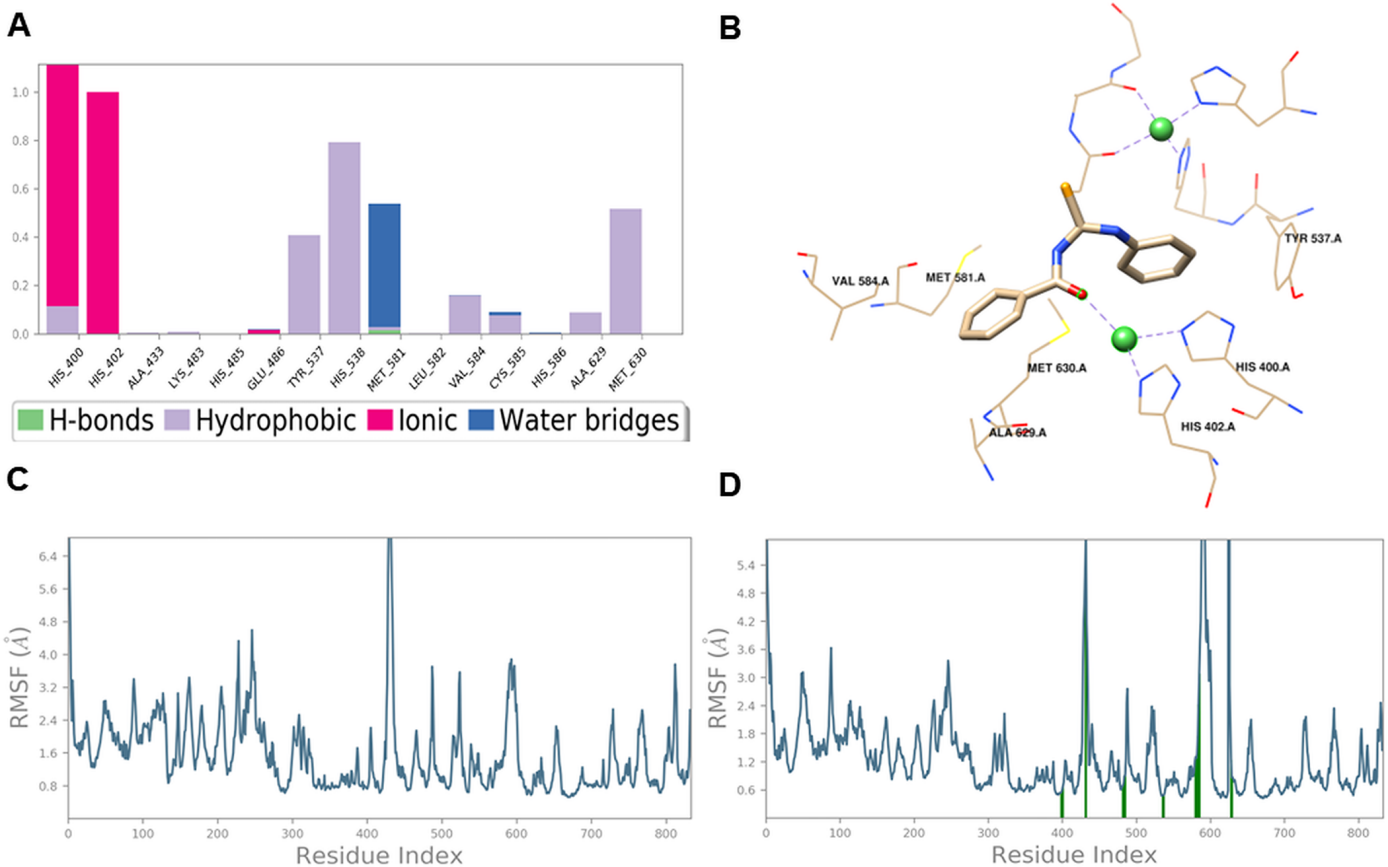
(A) Protein interactions with the inhibitor AF36. (B) Three-dimensional representation of AF36 bound to the allosteric site of *C. neoformans* urease. The ligand is shown in stick format, with the two Ni ions represented as green spheres. Coordination to the metal center is indicated by dashed lines. RMSF analysis of the native urease (C) and the AF36 complex (D) highlights increased fluctuations between residues Met581 and His600.

Derivative AF57, classified as a competitive inhibitor, exhibited a persistent coordination of its carbonyl group to the Ni center via the oxygen atom for the entire simulation time, resembling the binding mode observed for AF19 (Figure 6A). In contrast, AF55, identified as a mixed-type inhibitor, displayed a distinct inhibition profile, engaging in stable ionic interactions with Asp626 throughout the simulation and confirming its affinity for the allosteric site (Figure 6B). Furthermore, the close spatial relationship between the allosteric and catalytic sites enabled AF55 to maintain a simultaneous coordination of its carbonyl group with the Ni center for the full duration of the simulation (Figure 6C). These dual interaction modes may contribute to its mixed inhibition behavior and its ability to modulate urease activity through both direct catalytic site engagement and allosteric modulation. This is further supported by the RMSF profile (Figure 5D), which reveals noticeable fluctuations in both the catalytic and allosteric regions, indicating dynamic adjustments in these sites upon ligand binding.

**Figure 6:**
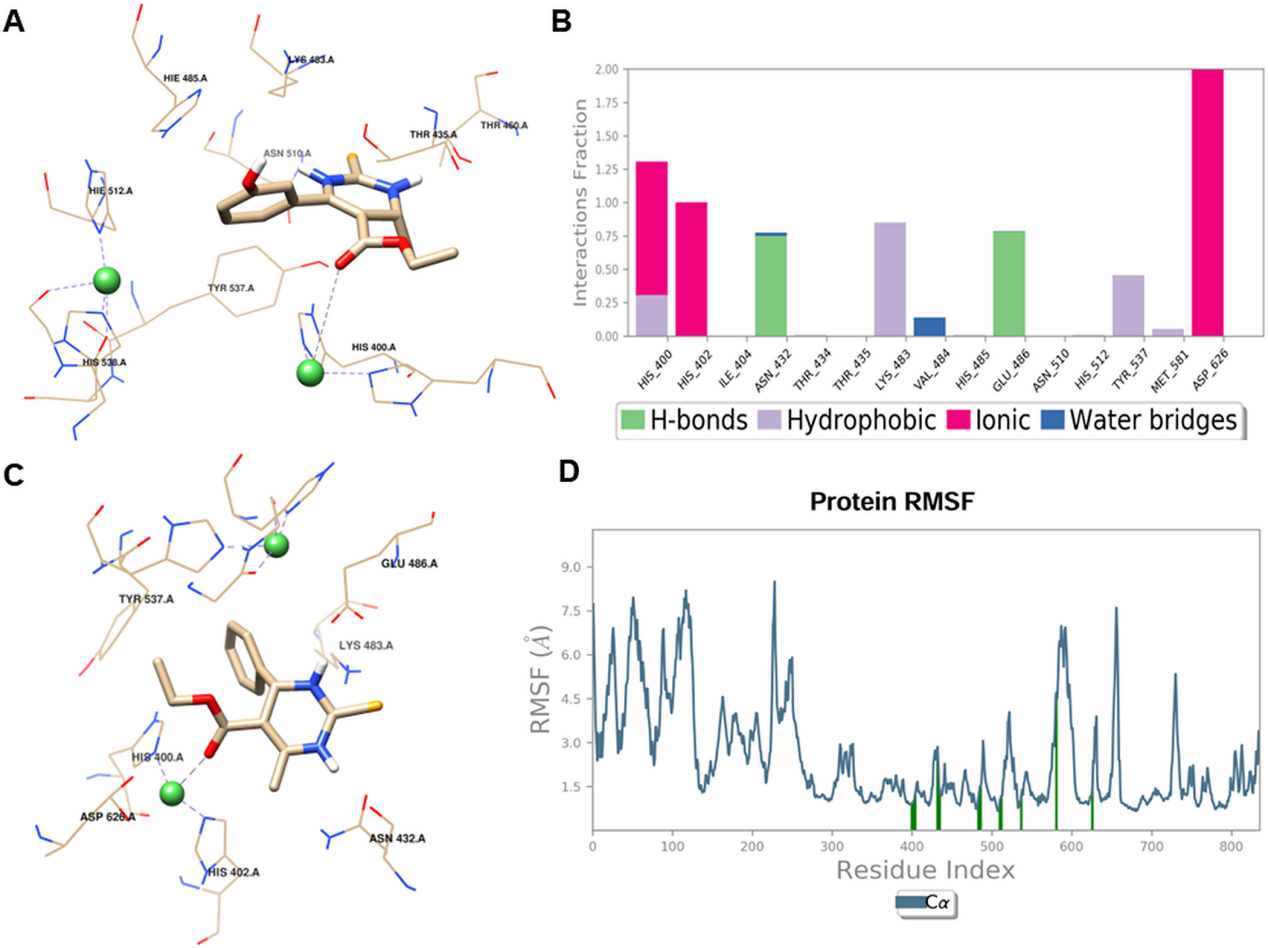
(A) Three-dimensional representation of AF57 bound to *C. neoformans* urease in the catalytic site, showing persistent coordination between the carbonyl group and the Ni center via the oxygen atom throughout the simulation. (B) Interaction profile of AF55, where bar heights represent the fraction of simulation time in which each interaction was maintained; values above 1.0 indicate multiple simultaneous contacts of the same type with the ligand. (C) Binding conformation of AF55 at the allosteric site, highlighting the coordination of its carbonyl group to the Ni center. (D) RMSF plot of the AF55–urease complex, with green vertical bars marking residues involved in ligand interactions, revealing noticeable fluctuations in both catalytic and allosteric regions.

### Pharmacokinetic and Toxicological Profiles *In Silico* Analysis of Novel Urease Inhibitors

The four lead urease inhibitors were evaluated for their pharmacokinetic and toxicological profiles using *in silico* predictions based on their chemical structures, providing ADMET (absorption, distribution, metabolism, excretion, and toxicity) data, guiding strategies to improve their pharmacological properties.^49^

Drug development prioritizes orally bioavailable molecules due to the advantages of oral administration.^50–53^ Lipinski’s rule of five was applied to assess oral bioavailability. These criteria include molecular weight <500 g/mol, TPSA <140 Å^2^, cLogP <5, less than 5 hydrogen bond donors, and less than 10 hydrogen bond acceptors, and all compounds met these parameters. In addition, the solubility coefficient (cLogS), ideally between −1 and −5 for adequate solubility and membrane permeability, ranged from −3.15 to −4.75 for the novel UREi (Table S12).

ADMET predictions showed that AF19, AF36, and AF55 may exhibit high intestinal permeability and good absorption, supporting potential oral use (Table 4). AF19 and AF36 exhibited moderate BBB permeability, and the data were consistent with the CNS permeability predictions. In contrast, AF55 and AF57 showed lower CNS distribution aligning with lower distribution volume and serum protein binding (Tables 4 and Table S14).

**Table 4:** *In Silico* Pharmacokinetic and Toxicity Parameters of Novel Urease Inhibitors Predicted by SwissADME and pkCSM^a^

		urease inhibitors			
parameters	PK and tox models	AF19	AF36	AF55	AF57
absorption	human intestinal absorption (% absorbed)^b^	92.15	91.75	66.25	55.48
	water solubility (log mol/L)	–3.96	–3.05	–2.58	–2.30
	Caco-2 permeability (log Papp × 10^–6^ cm/s)^c^	1.78	1.81	1.23	–0.01
distribution	VDss (log L/kg)^d^	0.46	–0.14	–0.02	–0.03
	BBB permeability (log BB)^e^	0.19	0.29	–0.01	–0.46
	CNS permeability (log PS)^f^	–1.52	–0.611	–2.55	–3.00
metabolism	CYP1A2 inhibition (yes/no)	yes	yes	no	no
	CYP2C19 inhibition (yes/no)	yes	yes	no	no
	CYP2C9 inhibition (yes/no)	yes	no	no	no
excretion	total clearance (log mL/min/kg)	0.10	1.66	1.85	1.90
toxicity	oral rat acute toxicity (LD_50_ in mol/kg)	2.03	2.29	2.78	2.57
	hepatotoxicity (yes/no)	no	no	no	no
	AMES toxicity (yes/no)	yes	no	no	no

a The complete ADMET profiles are available in Supporting Information Tables S13 and S14. VDss: volume of distribution; BBB: brain–blood barrier; CNS: central nervous system; AMES: mutagenic potential.

b <30% poorly absorbed.

c >0.9 high Caco-2 permeability.

d >0.45 high volume of distribution.

e >0.3 BBB permeability.

f >−2 penetrate the CNS.^58^

Regarding metabolism, AF19 and AF36 may inhibit several CYP enzymes, indicating potential drug–drug interactions, while AF55 and AF57 showed no inhibition of these enzymes. The CYP isoforms metabolize many drugs, and their inhibition may increase plasma concentrations and toxicity risk.^54–56^ Excretion predictions indicated high systemic clearance for most compounds, except AF19, suggesting prolonged exposure and longer half-life, which may reduce dosing frequency and improve adherence (Table 4 and Table S14).

Toxicity predictions revealed low to moderate acute oral toxicity for all compounds and an absence of hepatotoxicity, which is promising given the known hepatotoxic potential of some antifungal agents such as azole derivatives.^57^ Additional predictions indicated the absence of mutagenicity, tumorigenicity, irritation, and reproductive toxicity, supporting a favorable safety profile (Table 4 and Tables S13 and S14). All tested compounds showed no predicted toxicity in any of these categories, indicating a favorable safety profile.

### Urease Inhibitors Have No Toxicity in *Galleria mellonella* Larvae but Present Cytotoxicity on the Metastatic Breast Cancer Cells

All urease inhibitors, at 100 or 10.7 mg/kg (AF36), showed no toxicity in *G. mellonella* larvae with 100% larvae survival and high health index scores (Figure 7A,B) as well as dimethyl sulfoxide at 17.5% used to solubilize the UREi AF36. These data may corroborate with the *in silico* prediction that all leads showed low potential for toxicity (Table 4 and Table S13).

**Figure 7:**
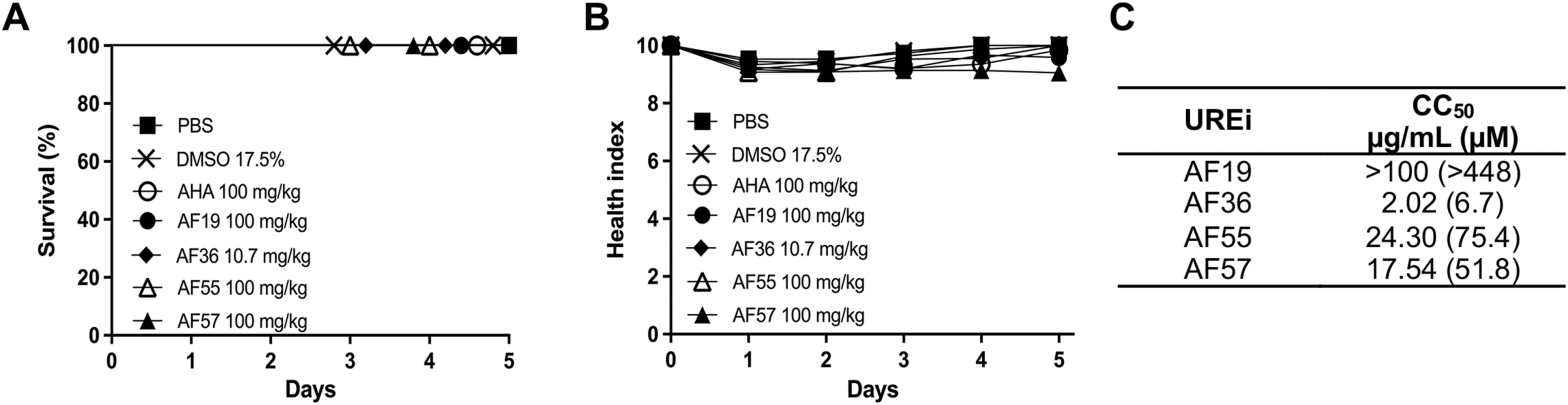
Toxicity of novel urease inhibitors on the invertebrate model of *Galleria mellonella* (A,B) and on mammalian cells (C). The survival curve (A) and morbidity curve (B) of larvae treated with 100 mg/kg of AHA, AF19, AF55, or AF57 and 10.7 mg/kg of AF36 were analyzed daily for 5 days at 35 °C (*n* = 20 larvae/group). The dimethyl sulfoxide control group was included (DMSO 17.5% in PBS) and only PBS was used as control groups (*n* = 20 larvae/group). (C) Cytotoxic concentration of 50% (CC_50_) of urease inhibitors in mammary adenocarcinoma cells (MCF-7) (average from technical duplicates and biological duplicates).

The UREi cytotoxicity was evaluated on the cells from metastatic breast cancer, and AF19 showed less toxic effect compared to other UREi (CC_50_ >448 μM). In contrast, selenium-containing compounds presented higher cytotoxicity, especially AF36 (CC_50_ = 6.7 μM) (Figure 7C). As previously described, monastrol is a potent anticancer agent^59^ and here, we also observed the cytotoxicity of its selenium analogs AF55 and AF57 (Figure 7C). However, the next generation of UREi must be tested against cancerous and noncancerous cell lines as well as vertebrate models to ensure that they are safe inhibitors or that they have dual biological action: antifungal and antineoplastic. This possibility cannot be excluded, as several drugs initially developed for one indication have later been repurposed for others.^60^

## Conclusions

A screening of new 34 synthetic urease inhibitors was assessed for their effects on cryptococcal growth and urease activity. Four lead candidates, AF19 (Schiff’s base), AF36 (benzoylselenourea), and AF55 and AF57 (Biginelli’s adducts derived from selenourea), exhibited low inhibitory concentration values, fungicidal activity, and nanomolar urease inhibition. All lead compounds reduced capsule thickness and increased membrane permeability, while AF55 and AF57 additionally inhibited melanin production. Kinetic studies combined with molecular docking and dynamic simulations suggested distinct mechanisms of interaction: AF19 and AF57 functioned as competitive nickel-coordinating inhibitors; AF36 exhibited noncompetitive inhibition through mobile-flap conformational modulation; and AF55 interacted with both catalytic and allosteric sites as a mixed-type inhibitor. Integrative analysis of the data highlights AF55 and AF57 as particularly promising anticryptococcal agents, combining a potent urease targeting, fungicidal activity, multivirulence factor inhibition, and favorable predicted toxicological and pharmacokinetic profiles.

## Materials and Methods

### Fungal Strains

*Cryptococcus neoformans* (strain H99 and 6 clinical isolates) and *Cryptococcus gattii* (strain R265 and 5 clinical isolates) were stored at −80 °C in Brain Hearth Infusion (BHI, Difco) supplemented with 20% glycerol in the culture collection from the Laboratory of Antifungal Chemotherapy (ICB/USP). The yeasts were recovered in Sabouraud dextrose agar (SDA, Difco) for 48 h at 35 °C and subcultured under the same conditions before experiments.

### Urease Inhibitors and Antifungals

56 synthetic compounds from chemical library of Dr. Ângelo de Fatima (IQ/UFMG) were previously designed to inhibit Jack bean urease enzyme and were tested for inhibition of urease and growth of *Cryptococcus*. Data from benzoylthioureas and benzoylselenoureas were reported previously by our research group,^20^ and here, we present novel data about 34 inhibitors (Table S1). After screening for antifungal activity, compounds from three chemical classes were selected: Schiff’s base,^21^ benzoylselenourea,^20^ and Biginelli adducts.^22^

Acetohydroxamic acid (AHA) was used as a classical urease inhibitor, and amphotericin B (AMB), fluconazole (FLC), and 5-fluorocytosine (5-FC) were used as standard antifungals (all from Sigma-Aldrich). All compounds, in the powder form, were aliquoted, and parts of them were dissolved in dimethyl sulfoxide (DMSO, Sigma-Aldrich) to obtain a stock solution maintained at −80 °C.

### Antifungal Activity of Urease Inhibitors on *Cryptococcus* Planktonic Cells

The urease inhibitors (UREi), in the range 0.25–128 μg/mL, were tested for the antifungal activity screening against *C. neoformans* H99 strain using the broth microdilution method.^61^ Briefly, the compounds were diluted in Roswell Park Memorial Institute 1640 medium buffered with 0.165 M 3-(*N*-morpholino)­propanesulfonic acid, pH 7.0 (= RMPI), and fungal suspension at 10^3^ CFU/mL was tested for 48 h at 35 °C to determine the minimum inhibitory concentration capable of inhibiting 50 and 90% of fungal growth (IC_50_ and IC_90_, respectively) by visual observation.

Additionally, to assess yeast urease activity of the compounds, Christensen broth (peptone 1 g/L, dextrose 1 g/L, sodium chloride 5 g/L, monobasic potassium phosphate 2 g/L, urea 20 g/L, and phenol red 12 mg/L) was used to determine the minimum inhibitory concentration of compound for urease activity (IC_URE_). This medium contains a pH indicator and appears orange under neutral conditions, turning pink under basic conditions. Therefore, the lowest concentration at which no color change was observed was defined as the IC_URE_.^20^

The minimum fungicidal concentration (MFC) was also determined by subculturing all samples in which the treatments led to the fungal growth inhibition in drug-free SDA at 35 °C for 48 h, and the MFC was defined as the lowest concentration of the compound able to reduce ≥99% viability of initial fungal inoculum.^62^ Finally, the lead molecules were selected for the next assays after antifungal screening on *C. neoformans* H99 according to the ICs, IC_URE_, and MFC values.

Subsequently, the selected lead molecules (AF19, AF36, AF55, and AF57) were combined with standard antifungals using the checkerboard assay to determine the fractional inhibitory concentration index (FICI).^63^ In addition, the lead molecules and antifungals were tested against clinical isolates of *C. neoformans* (*n* = 6) and *C. gattii* (*n* = 6).

### Antifungal Activity of Urease Inhibitors on *Cryptococcus* Biofilm

Antibiofilm activity of UREi was evaluated on biofilm formation, sessile, and dispersing cells from mature biofilm of *C. neoformans* H99 and *C. gattii* R265. Biofilms were formed by transferring 100 μL of the standardized suspension at 1 × 10^7^ CFU/mL in RPMI to the wells of 96-well flat-bottom polystyrene microplates and incubated at 35 °C, without shaking, for 72 h. The supernatant was collected to test the susceptibility of dispersed cells using broth microdilution protocol.^61^ Subsequently, sessile cells were washed 1× with PBS and treated with 100 μL of UREi (128 to 0.25 μg/mL) or AMB (8 to 0.015 μg/mL) solutions for 48 h at 35 °C, without agitation. In parallel, the yeasts were treated with UREi during biofilm formation at the same condition.^64^ The metabolic activity of biofilm cells was determined using the XTT reduction method^65^ and the SMIC values (minimum inhibitory concentration on sessile biofilm cells) were defined as the lowest concentration of the compound that inhibits 50% of the metabolic activity of sessile biofilm cells when compared to untreated cells. DMIC values (minimum inhibitory concentration on dispersion cells) were defined as the lowest concentration that inhibits 90% of fungal growth.

### Effect of Urease Inhibitors on *Cryptococcus* Capsule and Melanization

The yeasts of H99 and R265 strains were treated with the UREi at the subinhibitory concentration (0.5 × IC_90_) in RPMI for 48 h at 35 °C. The yeasts were stained with China ink and observed under a light microscope equipped with a camera to capture images (EVOS FL, Thermo Fisher Scientific). The capsule thickness was measured from 100 cells randomly using the ImageJ software (Image Processing and Analysis bundled with 64-bit Java 1.8.0 172).^36^

The effect of urease inhibitors on melanization was performed using a minimum medium (15 mM dextrose, 10 mM MgSO_4_, 29.4 mM KH_2_PO_4_, 13 mM glycine, 3 μM thiamine–HCl, 1% agar) supplemented with 1 mM L-3,4-dihydroxyphenylalanine (L-DOPA) to induce melanization in *C. neoformans* H99 and *C. gattii* R265.^36,66^ The medium with or without UREi, at concentration of 0.5 × IC_90_ or IC_90_, was added to a 24-well plate and 20 μL of fungal inoculum (1 × 10^6^ CFU/mL) was inoculated on top of the medium, incubated in a dark and humid chamber for 96 h at 35 °C.

### Time-Kill Curve

Before performing the assay, the technical limit of detection and drug carryover were determined according to Klepser et al.^38^ For the time-kill curve assay, yeasts from *C. neoformans* H99 or *C. gattii* R265 previously grown in Sabouraud dextrose broth for 48 h were standardized to 10^3^ CFU/mL, exposed to different concentrations of UREi (0.5 × IC_90_ to 8 × IC_90_) in RPMI, and incubated at 35 °C. After different incubation times, the samples were serially diluted (1:10) and 20 μL plated on Sabouraud dextrose agar. Plates were incubated at 35 °C for 48 h, and the colony-forming units were counted to calculate the Log CFU/mL.^38^

### Analysis of the Plasma Membrane Permeability

*C. neoformans* H99 and *C. gattii* R265 yeasts at 1 × 10 ^7^ CFU/mL were treated with UREi at IC_90_, MFC, and 4*x*MFC in PBS at 35 °C for different time points depending on the time-kill curve data: AF19 during 60 h for *C. neoformans* and 36 h for *C. gattii*; AF36 and AF55 for 2 h (both strains); and AF57 for 12 h (both strains). After, the cells adjusted to 3 × 10^6^ CFU/mL were exposed to 40 μg/mL of propidium iodide (PI, Invitrogen, Eugene, OR, USA) for 30 min in the dark at room temperature. The cells were analyzed using fluorescent microplate reader (BioTek Epoch 2) by measuring the absorbance at 493 nm.^36^

### Inhibitory Effect of the Compounds on the Cytoplasmic Urease from *Cryptococcus neoformans*

#### Crude Enzyme Obtention

*Cryptococcus neoformans* H99 yeasts were cultivated in 100 mL of Christensen broth for 72 h at 35 °C with agitation at 200 rpm. The culture was centrifuged (3900 × g/10 min) and washed with PBS three times to remove the culture medium. The lysate was obtained using the protocol previously described, and the total protein extract was freeze-dried.^20,67^

#### Urease Activity Assay

Prior to the assay, the freeze-dried crude protein extract was resuspended in 1 mL of assay buffer (10 mM sodium phosphate buffer, pH 7.0) and the concentration of total proteins was determined using the Bradford method.^68^ For the urease activity inhibition assay, in a 96-well microplate, 45 μL of the protein extract diluted in the assay buffer (1:8, with approximately 150–200 μg/mL of total proteins) was treated with 45 μL of the urease inhibitor (7 UREi: AHA, AF19, AF36, AF55–AF58) at concentrations of 1000, 250, 62.5, 16, 4, 1, 0.25, and 0.06 ng/mL. Urea (10 μL at 100 mM) was used as substrate and the reaction was maintained for 10 min under agitation at 600 rpm, 25 °C.^41^ Then, 45 μL of solution A (1% phenol; 0.005% sodium nitroprusside) and 70 μL of solution B (0.5% sodium hydroxide; 0.1% sodium hypochlorite) were added to all wells and incubated for 5 min at 50 °C under agitation at 600 rpm. Urease activity was quantified by colorimetric assay measuring the ammonia production (μmol/min/mg total protein) at 670 nm.^69^ The inhibitory concentration capable of inhibiting 50% of urease activity (ureIC_50_) was determined by linear regression calculation in GraphPad Prism 10.0.

#### Urease Inhibition Kinetic

Urease from the crude protein extract was used to determine the steady-state kinetics. First, the urease activity was determined as described above using different urea concentrations (25, 50, 100, 200, and 400 mM) [*S*]. The reaction initial velocity (*V_o_*) was measured considering the ammonia production (μmol/min/mg total protein) for each urea concentration and a nonlinear regression analysis used to generate estimates of the Michaelis–Menten kinetics parameters and calculate the Michaelis constant (*K_m_*) and the maximum velocity (*V*_max_)^70^ expressed in the eq 1:

$$V_{o}=\frac{V_{\max}\left[S\right]}{K_{m}+\left[S\right]}$$ 1

The enzymatic kinetic was performed with the selected UREi (AHA, AF19, AF36, AF55, and AF57) using the fixed protein extract dilution (1:8, 150–200 μg/mL of total protein) varying the urea concentration (10, 25, 50 100, 200, and 400 mM) and inhibitor concentration based on the ureIC_50_ obtained previously (0.25 × ureIC_50_ to 4 × ureIC_50_). The reaction was maintained for 10 min under agitation at 600 rpm, 25 °C. The reaction was stopped and absorbance measured as described before. The ammonia production (μmol/min/mg total protein) used as initial reaction velocity (*V_o_*) was applied to identify the inhibition type and UREi inhibition constants on *C. neoformans* urease. The inverse of initial reaction velocity (1/*V_o_*) was plotted versus the reciprocal of the substrate concentration (1/[S]) on a Lineweaver–Burk (LB) graph (eq 2). The Michaelis constant (*K_m_*) and maximum reaction velocity (*V*_max_) were calculated to determine the type of inhibition on urease by each inhibitor tested:^45^

$$\frac{1}{V_{o}}=\frac{K_{m}}{V_{\max}\left[S\right]}+\frac{1}{V_{\max}}$$ 2

The competitive, noncompetitive, and mixed-type inhibition constants (K_i_)—the dissociation constant of the enzyme by the inhibitor [*I*]—were derived from secondary LB plots using eqs 3, 4, and 5, respectively, calculated using GraphPad Prism v.10. Values of appK_m_, and app*V*_max_ represent the apparent kinetic parameters in the presence of inhibitors.

$$V_{O}=\frac{V_{\max}[S]}{\mathrm{app}K_{m}+[S]}\mathrm{where}\;\mathrm{app}K_{m}=K_{m}\left(\frac{1+[I]}{K_{i}}\right)$$ 3

$$V_{o}=\frac{\mathrm{app}V_{\max}[S]}{K_{m}+[S]}\mathrm{where}\;\mathrm{app}V_{\max}=\frac{V_{\max}}{\left(\frac{1+I}{K_{i}}\right)}$$ 4

$$V_{o}=\frac{\mathrm{app}V_{\max}[S]}{\mathrm{app}K_{m}+[S]}\;\mathrm{where}\;\mathrm{app}V_{\max}=\frac{V_{\max}}{\left(\frac{1+I}{\alpha^{*}K_{i}}\right)}\;\mathrm{and}\;\mathrm{app}K_{m}=\frac{K_{m}\left(\frac{1+I}{K_{i}}\right)}{\left(\frac{1+I}{\alpha^{*}K_{i}}\right)}$$ 5

$$\alpha =\frac{K_{\mathrm{app}}}{K_{m}};K_{i}=\frac{\left[I\right]}{\left(\alpha -1\right)}$$

### Molecular Docking and Dynamic Simulations of Urease Inhibitors using the AlphaFold Prediction Structure of *Cryptococcus neoformans* Urease

Molecular docking studies were conducted to investigate the interaction profiles of the selected compounds with *C. neoformans* urease AlphaFold v.2 predicted structure. The workflow encompassed the generation and validation of a three-dimensional homology model of the enzyme, followed by molecular docking to predict the most favorable binding poses and molecular dynamics simulations to assess the stability of the protein–ligand complexes over time. All computational procedures, including model construction, refinement, docking protocols, and simulation parameters, were performed according to methodologies previously described by our research group.^20^

### Absorption, Distribution, Metabolism, Excretion, and Toxicity (ADMET) *In Silico* Analysis of Urease Inhibitors

SwissADME (https://www.swissadme.ch/), developed by the Swiss Institute of Bioinformatics,^71^ and pkCSM (https://biosig.lab.uq.edu.au/pkcsm/prediction),^58^ developed by the Biosig Lab at the University of Queensland, were employed as *in silico* tools for the prediction of the absorption, distribution, metabolism, excretion, and toxicity (ADMET) of the UREi (AF19, AF36, AF55, and AF57). The molecular structures of compounds were drawn using a SwissADME and subsequently converted into canonical SMILES (simplified molecular input line entry system) format (Table S11). These SMILES representations were then submitted to both SwissADME and pkCSM for the ADMET profiling. Additionally, key molecular features related to druglikeness were evaluated using the Molinspiration online platform (http://www.molinspiration.com/).

### Toxicity Analysis

#### Cytotoxicity in Mammalian Cells

Cells from metastatic breast cancer (MCF-7) were seeded in 96-well plates at 6 × 10^3^ cells per well in 200 μL of Dulbecco’s modified Eagle medium supplemented with Nutrient Mixture F12 (DMEM-F12) with 10% fetal bovine serum and incubated for 24 h at 37 °C and 5% CO_2_ to obtain a monolayer. The cells were treated with different concentrations of compounds (0.0064 to 100 μg/mL) for 72 h at 37 °C and 5% CO_2_. After, the cell viability was determined using MTT assay to determine the cytotoxic concentration of 50% (CC_50_) values and their 95% confidence interval.^72^

#### Galleria Mellonella Model

Larvae ranging from 2.0 to 2.5 cm in length and 150–200 mg in body mass were used to determine the toxicity of the UREi diluted in PBS at 100 mg/kg, except for the AF36 tested, which was 10.7 mg/kg due to its lower aqueous solubility. The solutions were injected (10 μL) into the last left pro-leg of the larvae (20 larvae *per* group). Furthermore, PBS was used as a group control of mechanical injury and vehicle, and DMSO control group of the maximal dose (17.5%) was also included. The larvae were maintained at 35 °C, and the survival and health index of all groups was monitored daily for 5 days as previously described.^36^

### Statistical Analysis

The statistical analyses were performed using the GraphPad Prism 10.0, and *p* values of less than 0.05 were considered statistically significant.

## Supplementary Material



## Data Availability

All data are available throughout the paper and supporting files. Raw data (tables, figures, and pictures) will be made available from the corresponding author upon request.

## Notes

Antifungal screening of new urease inhibitors in different culture media on *Cryptococcus neoformans* H99 strain (Table S1); susceptibility of clinical isolates of *Cryptococcus neoformans* and *Cryptococcus gattii* to novel urease inhibitors (AF19, AF36, AF55, and AF57) and standard antifungals in different culture media (Tables S2–S9); kinetic parameters of urease obtained from crude protein extract and inhibited by urease inhibitors (Table S10); *in silico* pharmacokinetic and toxicity parameters of novel urease inhibitors (Tables S11–S13); *Cryptococcus neoformans* H99 urease kinetics plots of Michaelis–Menten (A) and Lineweaver–Burk (B) (Figure S1); Lineweaver–Burk double-reciprocal linear fitting of *Cryptococcus neoformans* urease from crude extract inhibited by urease inhibitors (Figure S2) (PDF)
