# Supplementary material for: Novel Inhibitors Targeting Urease Promote Fungicidal and Antivirulence Effects in Cryptococcus
Source: ACS Omega. 2026 Jul 1;11(27):40601–16. doi: 10.1021/acsomega.6c03740 (PMC13382658; doi:10.1021/acsomega.6c03740)
Supplement: Supplementary file 1 [file ao6c03740_si_001.pdf]

Supplementary information

Title: Novel inhibitors targeting urease promote fungicidal and anti-virulence effects in *Cryptococcus*.

Authors: *Thayná Lopes Barreto[a], Nathália Evelyn Morais Costa[b], Nathalia Monteiro Lins Freire[c], Larissa Costa de Almeida[d], Rodrigo dos Anjos Miguel[d], Leticia Veras Costa-Lotufo[d], Gislaine da Silva-Rodrigues[a], Cleiton Moreira da Silva[b], Thiago Mendonça de Aquino[c], Eduardo Eliezer Alberto[b], Ângelo de Fátima[b,e], Kelly Ishida[a*]*

Author Address: [a]Department of Microbiology, Institute of Biomedical Sciences, University of São Paulo, 1374 Prof. Lineu Prestes Avenue, São Paulo, SP 05508-000, Brazil; [b]Department of Chemistry, Institute of Exact Sciences, Federal University of Minas Gerais, 6627 Pres. Antônio Carlos Avenue, Belo Horizonte, MG 31270-901, Brazil; [c]Research Group on Therapeutic Strategies - GPET, Institute of Chemistry and Biotechnology, Federal University of Alagoas, Lourival Melo Mota Avenue, Maceió, AL 57072-900, Brazil; [d]Department of Pharmacology, Institute of Biomedical Sciences, University of São Paulo, 1524 Prof. Lineu Prestes Avenue, São Paulo, SP 05508-000, Brazil

***Corresponding Author:** E-mail: ishidakelly@usp.br; Phone: +55(11)30917204

Table S1. Susceptibility of *Cryptococcus neoformans* H99 to urease inhibitors in different culture media. Minimum Inhibitory Concentration values (IC_{50} and IC_{90}: 50% and 90% inhibition of fungal growth, respectively), Minimum Inhibitory Concentration of Urease Activity (IC_{URE}) and Minimum Fungicidal Concentration (MFC) in RPMI and Christensen broth. AHA: acetohydroxamic acid, THIO: thiourea.

Molecules	Structures	Media	***Cryptococcus neoformans* H99** Range [µM (µg/mL)]			
			IC_{50}	IC_{90}	IC_{URE}	MFC
AHA	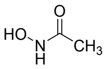	RPMI	1,705 (128)	1,705 (128)	ND	>1,705 (>128)
	Chemical Formula: $C_2H_5NO_2$ Molecular Weight: 75.0670	Christensen	852-1,705 (64-128)	1,705 (128)	1,705 (128)	>1,705 (>128)
THIO	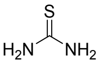	RPMI	1,682 (128)	>1,682 (>128)	ND	>1,682 (>128)
	Chemical Formula: CH_4N_2S Molecular Weight: 76.1170	Christensen	210-1,682 (16-128)	420-1,682 (32-128)	>1,682 (>128)	>1,682 (>128)
**Imidazoles and Benzothiazoles**						
AF1	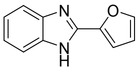	RPMI	695 (128)	>695 (>128)	ND	>695 (>128)
	Chemical Formula: $C_{11}H_8N_2O$ Molecular Weight: 184.1980	Christensen	347-695 (64-128)	695 (128)	695 (128)	695 (128)
AF2	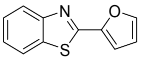	RPMI	318-636 (64-128)	636 (128)	ND	>636 (>128)
	Chemical Formula: $C_{11}H_7NOS$ Molecular Weight: 201.2430	Christensen	159-318 (32-64)	318 (64)	318 (64)	636 (128)
AF3	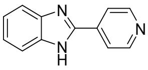	RPMI	656 (128)	>656 (>128)	ND	>656 (>128)
	Chemical Formula: $C_{12}H_9N_3$ Molecular Weight: 195.2250	Christensen	328-656 (64-128)	656 (128)	656 (128)	>656 (>128)
AF4	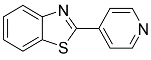	RPMI	301 (64)	603 (128)	ND	603 (128)
	Chemical Formula: $C_{12}H_8N_2S$ Molecular Weight: 212.2700	Christensen	301 (64)	301 (64)	301 (64)	603 (128)
**Acylbenzoylthioureas[a]**						
AF5	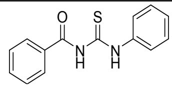	RPMI	>499 (>128)	>499 (>128)	ND	>499 (>128)
	Chemical Formula: $C_{14}H_{12}N_2OS$ Molecular Weight: 256.3230	Christensen	499 (128)	>499 (>128)	>499 (>128)	>499 (>128)
AF6	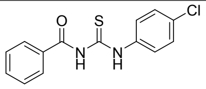	RPMI	>440 (>128)	>440 (>128)	ND	>440 (>128)
	Chemical Formula: $C_{14}H_{11}N_2OSCl$ Molecular Weight: 290.7650	Christensen	220-440 (64-128)	440 (128)	220-440 (64-128)	>440 (>128)
AF7	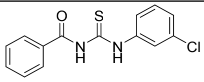	RPMI	>440 (>128)	>440 (>128)	ND	>440 (>128)
	Chemical Formula: $C_{14}H_{11}N_2OSCl$ Molecular Weight: 290.7650	Christensen	440 (128)	440 (128)	440 (128)	>440 (>128)
AF8	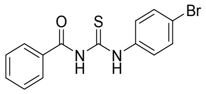	RPMI	>382 (>128)	>382 (>128)	ND	>382 (>128)
	Chemical Formula: $C_{14}H_{11}N_2OSBr$ Molecular Weight: 335.2190	Christensen	382 (128)	>382 (>128)	382 (128)	>382 (>128)

AF9	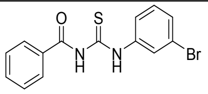 Chemical Formula: $C_{14}H_{11}N_2OSBr$ Molecular Weight: 335.2190	RPMI	>382 (>128)	>382 (>128)	ND	>382 (>128)
		Christensen	382 (128)	382 (128)	382 (128)	>382 (>128)
			**Guanidines**			
AF10	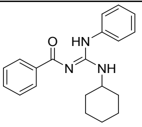 Chemical Formula: $C_{20}H_{23}N_3O$ Molecular Weight: 321.4240	RPMI	199-398 (64-128)	398 (128)	ND	>398 (>128)
		Christensen	99-199 (32-64)	99-398 (32-128)	99-398 (32-128)	>398 (>128)
AF11	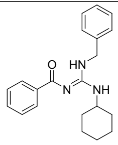 Chemical Formula: $C_{21}H_{25}N_3O$ Molecular Weight: 335.4510	RPMI	>381 (>128)	>381 (>128)	ND	>381 (>128)
		Christensen	381 (128)	>381 (>128)	>381 (>128)	>381 (>128)
			**Biginelli adducts[b]**			
AF12	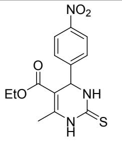 Chemical Formula: $C_{14}H_{15}N_3O_4S$ Molecular Weight: 321.3510	RPMI	>398 (>128)	>398 (>128)	ND	>398 (>128)
		Christensen	>398 (>128)	>398 (>128)	>398 (>128)	>398 (>128)
AF13	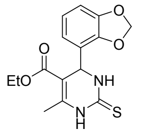 Chemical Formula: $C_{15}H_{16}N_2O_4S$ Molecular Weight: 320.3630	RPMI	>399 (>128)	>399 (>128)	ND	>399 (>128)
		Christensen	>399 (>128)	>399 (>128)	>399 (>128)	>399 (>128)
AF14	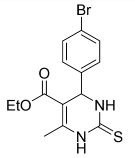 Chemical Formula: $C_{14}H_{15}N_2O_2SBr$ Molecular Weight: 355.2500	RPMI	180-360 (64-128)	>360 (>128)	ND	>360 (>128)
		Christensen	90-360 (32-128)	180-360 (64-128)	90-360 (32-128)	360 (128)
AF15	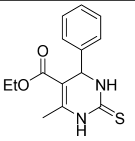 Chemical Formula: $C_{14}H_{16}N_2O_2S$ Molecular Weight: 276.3540	RPMI	>463 (>128)	>463 (>128)	ND	>463 (>128)
		Christensen	232-463 (64-128)	463 (128)	463 (128)	>463 (>128)
			**Schiff's bases[c]**			
AF16	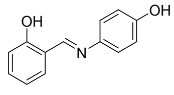 Chemical Formula: $C_{13}H_{11}NO_2$ Molecular Weight: 213.2360	RPMI	150-300 (32-64)	300-600 (64-128)	ND	600 (128)
		Christensen	75-300 (16-64)	150-600 (32-128)	75-600 (16-128)	150-600 (32-128)
AF17	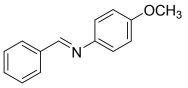 Chemical Formula: $C_{14}H_{13}NO$ Molecular Weight: 211.2640	RPMI	>606 (>128)	>606 (>128)	ND	>606 (>128)
		Christensen	606 (128)	>606 (>128)	>606 (>128)	>606 (>128)
**AF18**	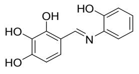 Chemical Formula: $C_{13}H_{11}NO_4$ Molecular Weight: 245.2340	RPMI	33 (8)	33 (8)	ND	65-261 (16-64)
		Christensen	8-33 (2-8)	16-33 (4-8)	8-33 (2-8)	33-130 (8-32)

**AF19**	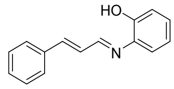	RPMI	9-18 (2-4)	9-18 (2-4)	ND	9-72 (2-16)
	Chemical Formula: $C_{15}H_{13}NO$<br>Molecular Weight: 223.2750	Christensen	9-18 (2-4)	9-18 (2-4)	4-18 (1-4)	9-72 (2-16)
**AF20**	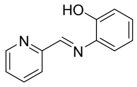	RPMI	20-81 (4-16)	20-161 (4-32)	ND	40-323 (8-64)
	Chemical Formula: $C_{12}H_{10}N_2O$<br>Molecular Weight: 198.2250	Christensen	10-40 (2-8)	20-40 (4-8)	10-40 (2-8)	81-161 (16-32)
**AF21**	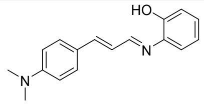	RPMI	30-120 (8-32)	60-240 (16-64)	ND	60-480 (16-128)
	Chemical Formula: $C_{17}H_{18}N_2O$<br>Molecular Weight: 266.3440	Christensen	15-60 (4-16)	30-120 (8-32)	15-60 (4-16)	60-480 (16-128)
			**Usnic acids**			
AF22	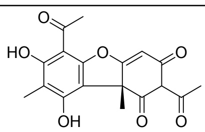	RPMI	186-372 (64-128)	186-372 (64-128)	ND	186-372 (64-128)
	Chemical Formula: $C_{18}H_{16}O_7$<br>Molecular Weight: 344.3190	Christensen	93-186 (32-64)	186 (64)	186 (64)	93-372 (32-128)
AF23	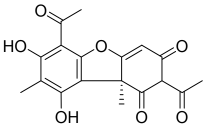	RPMI	93-372 (32-64)	186-372 (64-128)	ND	186-372 (64-128)
	Chemical Formula: $C_{18}H_{16}O_7$<br>Molecular Weight: 344.3190	Christensen	46-186 (16-64)	93-372 (32-128)	93-372 (32-64)	93-372 (32-128)
			**Isothiocyanate**			
AF24	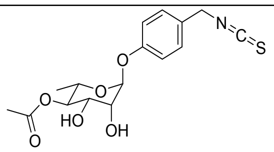	RPMI	182-363 (64-128)	363 (128)	ND	363 (128)
	Chemical Formula: $C_{16}H_{18}O_6NS$<br>Molecular Weight: 352.3800	Christensen	91-182 (32-64)	182 (64)	91-182 (32-64)	363 (128)
			**Benzoylthioureas[a]**			
AF26	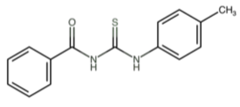	RPMI	>473 (>128)	>473 (>128)	ND	>473 (>128)
	Chemical Formula: $C_{15}H_{14}N_2OS$<br>Molecular Weight: 270.3500	Christensen	118 (32)	237 (64)	≤0.9 (≤0.25)	>473 (>128)
**AF27**	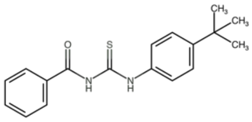	RPMI	26 (8)	51 (16)	ND	>410 (>128)
	Chemical Formula: $C_{18}H_{20}N_2OS$<br>Molecular Weight: 312.4310	Christensen	3 (1)	6 (2)	1.6 (0.5)	410 (128)
AF32	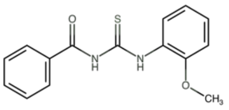	RPMI	>447 (>128)	>447 (>128)	ND	>447 (>128)
	Chemical Formula: $C_{15}H_{14}N_2O_2S$<br>Molecular Weight: 286.3490	Christensen	223 (64)	447 (128)	112 (32)	>447 (>128)
**AF33**	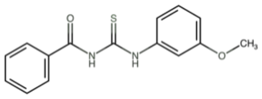	RPMI	112 (32)	223 (64)	ND	>447 (>128)
	Chemical Formula: $C_{15}H_{14}N_2O_2S$<br>Molecular Weight: 286.3490	Christensen	14 (4)	28 (8)	14 (4)	112 (32)
AF34	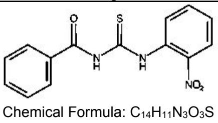	RPMI	>425 (>128)	>425 (>128)	ND	>425 (>128)
	Chemical Formula: $C_{14}H_{11}N_3O_3S$<br>Molecular Weight: 301.3200	Christensen	>425 (>128)	>425 (>128)	>425 (>128)	>425 (>128)
AF35		RPMI	>425	>425	ND	>425

			(>128)	(>128)		(>128)
	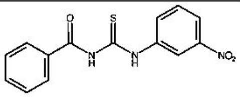 Chemical Formula: $C_{14}H_{11}N_3O_3S$ Molecular Weight: 301.3200	Christensen	>425 (>128)	>425 (>128)	>425 (>128)	>425 (>128)
		**Benzoylselenoureas[d]**				
**AF36**	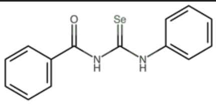 Chemical Formula: $C_{14}H_{12}N_2OSe$ Molecular Weight: 303.2340	RPMI	7 (2)	7 (2)	ND	7 (2)
		Christensen	0.2 (0.06)	0.4 (0.12)	0.2 (0.06)	0.4 (0.12)
**AF37**	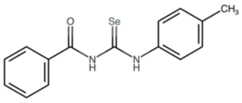 Chemical Formula: $C_{15}H_{14}N_2OSe$ Molecular Weight: 317.2610	RPMI	50 (16)	50 (16)	ND	50 (16)
		Christensen	1.5 (0.5)	1.5 (0.5)	1.5 (0.5)	3 (1)
AF38	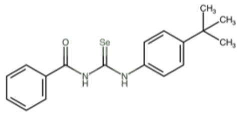 Chemical Formula: $C_{18}H_{20}N_2OSe$ Molecular Weight: 359.3420	RPMI	>356 (>128)	>356 (>128)	ND	>356 (>128)
		Christensen	>356 (>128)	>356 (>128)	>356 (>128)	>356 (>128)
**AF39**	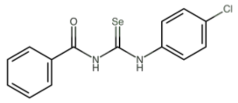 Chemical Formula: $C_{14}H_{11}N_2OSeCl$ Molecular Weight: 337.6760	RPMI	47 (16)	47 (16)	ND	47 (16)
		Christensen	1.5 (0.5)	1.5 (0.5)	0.7 (0.25)	1.5 (0.5)
**AF40**	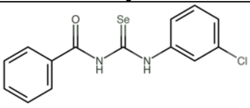 Chemical Formula: $C_{14}H_{11}N_2OSeCl$ Molecular Weight: 337.6760	RPMI	24 (8)	47 (16)	ND	47 (16)
		Christensen	0.7 (0.25)	0.7 (0.25)	0.3 (0.12)	0.7 (0.25)
AF41	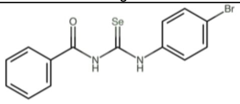 Chemical Formula: $C_{14}H_{11}N_2OSeBr$ Molecular Weight: 382.1300	RPMI	335 (128)	>335 (>128)	ND	>335 (>128)
		Christensen	21 (8)	42 (16)	21 (8)	42 (16)
**AF42**	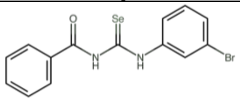 Chemical Formula: $C_{14}H_{11}N_2OSeBr$ Molecular Weight: 382.3000	RPMI	10 (4)	10 (4)	ND	10 (4)
		Christensen	0.3 (0.12)	0.6 (0.25)	0.2 (0.06)	0.6 (0.25)
**AF43**	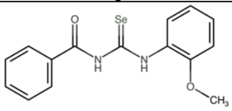 Chemical Formula: $C_{15}H_{14}N_2O_2Se$ Molecular Weight: 333.2600	RPMI	24 (8)	24 (8)	ND	24 (8)
		Christensen	1.5 (0.5)	1.5 (0.5)	1.5 (0.5)	1.5 (0.5)
**AF44**	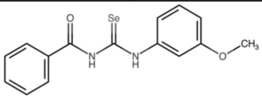 Chemical Formula: $C_{15}H_{14}N_2O_2Se$ Molecular Weight: 333.2600	RPMI	3 (1)	3 (1)	ND	3 (1)
		Christensen	0.4 (0.12)	0.4 (0.12)	0.4 (0.12)	1.5 (0.5)
**AF45**	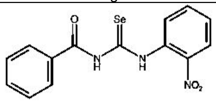 Chemical Formula: $C_{14}H_{11}N_3O_3Se$ Molecular Weight: 348.2310	RPMI	23 (8)	46 (16)	ND	46 (16)
		Christensen	3 (1)	3 (1)	3 (1)	6 (2)
AF46	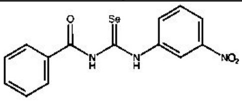 Chemical Formula: $C_{14}H_{11}N_3O_3Se$ Molecular Weight: 348.2310	RPMI	92 (32)	184 (64)	ND	184 (64)
		Christensen	3 (1)	3 (1)	3 (1)	3 (1)
		**Biginelli adducts[e]**				
AF47	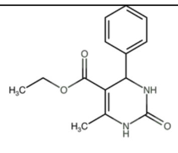 Chemical Formula: $C_{14}H_{15}N_2O_3$ Molecular Weight: 259.28	RPMI	>494 (>128)	>494 (>128)	ND	>494 (>128)
		Christensen	>494 (>128)	>494 (>128)	>494 (>128)	>494 (>128)

AF48	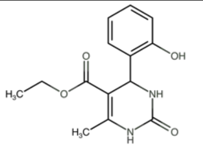 Chemical Formula: $C_{14}H_{15}N_2O_4$ Molecular Weight: 275.28	RPMI	>465 (>128)	>465 (>128)	ND	>465 (>128)
		Christensen	>465 (>128)	>465 (>128)	>465 (>128)	>465 (>128)
AF49	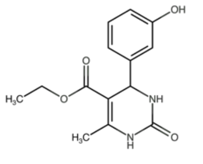 Chemical Formula: $C_{14}H_{15}N_2O_4$ Molecular Weight: 275.28	RPMI	>465 (>128)	>465 (>128)	ND	>465 (>128)
		Christensen	>465 (>128)	>465 (>128)	>465 (>128)	>465 (>128)
AF50	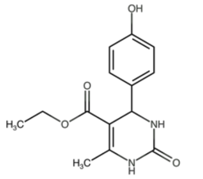 Chemical Formula: $C_{14}H_{15}N_2O_4$ Molecular Weight: 275.28	RPMI	>465 (>128)	>465 (>128)	ND	>465 (>128)
		Christensen	>465 (>128)	>465 (>128)	>465 (>128)	>465 (>128)
AF52	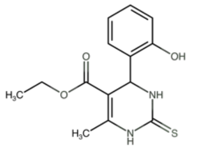 Chemical Formula: $C_{14}H_{15}N_2O_3S$ Molecular Weight: 291.34	RPMI	>439 (>128)	>439 (>128)	ND	>439 (>128)
		Christensen	>439 (>128)	>439 (>128)	>439 (>128)	>439 (>128)
AF53	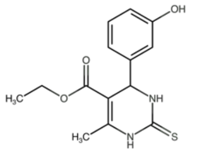 Chemical Formula: $C_{14}H_{15}N_2O_3S$ Molecular Weight: 291.34	RPMI	>439 (>128)	>439 (>128)	ND	>439 (>128)
		Christensen	>439 (>128)	>439 (>128)	>439 (>128)	>439 (>128)
AF54	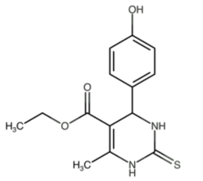 Chemical Formula: $C_{14}H_{15}N_2O_3S$ Molecular Weight: 291.34	RPMI	>439 (>128)	>439 (>128)	ND	>439 (>128)
		Christensen	>439 (>128)	>439 (>128)	>439 (>128)	>439 (>128)
**AF55**	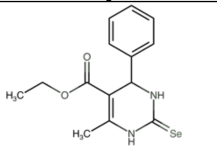 Chemical Formula: $C_{14}H_{15}N_2O_2Se$ Molecular Weight: 322.24	RPMI	12 (4)	12 (4)	ND	12 (4)
		Christensen	0.7 (0.25)	1.5 (0.5)	0.7 (0.25)	1.5 (0.5)
**AF56**	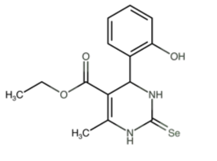 Chemical Formula: $C_{14}H_{15}N_2O_3Se$ Molecular Weight: 338.24	RPMI	12 (4)	12 (4)	ND	12 (4)
		Christensen	0.7 (0.25)	1.5 (0.5)	0.7 (0.25)	1.5 (0.5)
**AF57**	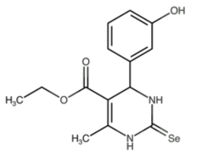 Chemical Formula: $C_{14}H_{15}N_2O_3Se$ Molecular Weight: 338.24	RPMI	6 (2)	6 (2)	ND	12 (4)
		Christensen	0.7 (0.25)	0.7 (0.25)	0.7 (0.25)	0.7 (0.25)

**AF58**	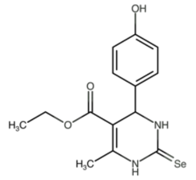 Chemical Formula: $C_{14}H_{15}N_2O_3Se$ Molecular Weight: 338.24	RPMI	24 (8)	48 (16)	ND	48 (16)
		Christensen	3 (1)	3 (1)	3 (1)	3 (1)
AF59	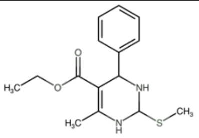 Chemical Formula: $C_{15}H_{18}N_2O_2S$ Molecular Weight: 290.38	RPMI	>441 (>128)	>441 (>128)	ND	>441 (>128)
		Christensen	>441 (>128)	>441 (>128)	>441 (>128)	>441 (>128)
AF60	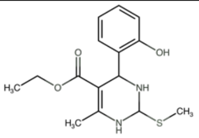 Chemical Formula: $C_{15}H_{18}N_2O_3S$ Molecular Weight: 306.38	RPMI	>418 (>128)	>418 (>128)	ND	>418 (>128)
		Christensen	>418 (>128)	>418 (>128)	>418 (>128)	>418 (>128)
AF61	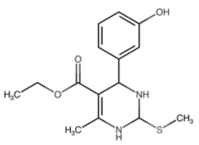 Chemical Formula: $C_{15}H_{18}N_2O_3S$ Molecular Weight: 306.38	RPMI	>418 (>128)	>418 (>128)	ND	>418 (>128)
		Christensen	>418 (>128)	>418 (>128)	>418 (>128)	>418 (>128)
AF62	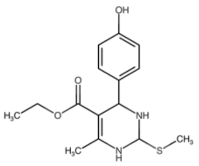 Chemical Formula: $C_{15}H_{18}N_2O_3S$ Molecular Weight: 306.38	RPMI	>418 (>128)	>418 (>128)	ND	>418 (>128)
		Christensen	>418 (>128)	>418 (>128)	>418 (>128)	>418 (>128)

ND: not determined

[a] Brito, T. O.; Souza, A. X.; Mota, Y. C. C.; Morais, V. S. S.; De Souza, L. T.; De Fátima, A. et al. Design, syntheses and evaluation of benzoylthioureas as urease inhibitors of agricultural interest. *RSC Advances,* **2015**, *5* (55), 44507-44515. DOI: 10.1039/C5RA07886E

[b] Braga, T. C.; Silva, T. M.; Maciel, T. M. S.; Da Silva, E. C. D.; Silva-Júnior, E. F.; Modolo, L. V. et al. Ionic liquid-assisted synthesis of dihydropyrimidin(thi)one Biginelli Adducts and investigation of their mechanism of urease inhibition. *New Journal of Chemistry*, **2019**, *43* (38), 15187– 15200. DOI: 10.1039/C9NJ03556G

[c] Da Silva, M. C.; Silva, M. M.; Reis, F. S.; Ruiz, A. L. T. G.; Carvalho, J. E. Santos, J. C. C. et al. Studies on free radical scavenging, cancer cell antiproliferation, and calf thymus DNA interaction of Schiff bases. *Journal of Photochemistry & Photobiology B: Biology,* **2017**, *172,* 129–138. DOI: 10.1016/j.jphotobiol.2017.05.020

[d] Costa, N. E. M.; Barreto, T. L.; Freire, N. M. L.; Silva, J. C. S.; Aquino, T. M.; Alberto, E. E. et al. Benzoylselenoureas: A novel dual-action inhibitor targeting fungal growth and urease activity in *Cryptococcus neoformans. ACS Omega*, **2025,** *10* (37), 43120-43128. DOI: 10.1021/acsomega.5c06398

[e] Costa, N. E. M. Inibição da urease como ferramenta medicinal e agrícola: a influência de átomos de selênio e boro na atividade antiureolítica de inibidores da urease conhecidos. Ph.D. thesis, Federal University of Minas Gerais, 2024. https://repositorio.ufmg.br/bitstreams/bda309a9-2e97-44f9-ab46-17794200603d/download

Table S2. Susceptibility of clinical isolates of *Cryptococcus neoformans* to novel urease inhibitors AF19 and AF36 in different culture media. Minimum Inhibitory Concentration values (IC_{50} and IC_{90}: 50% and 90% inhibition of fungal growth, respectively), Minimum Inhibitory Concentration of Urease Activity (IC_{URE}) and Minimum Fungicidal Concentration (MFC) were performed in RPMI and Christensen broth and are expressed in both in μM and μg/mL (in parenthesis). ND: not determined.

Clinical isolates	Media	AF19				AF36			
		IC_{50}	IC_{90}	IC_{URE}	MFC	IC_{50}	IC_{90}	IC_{URE}	MFC
H99	RPMI	9-18 (2-4)	9-18 (2-4)	ND	9-72 (2-16)	7-53 (2-16)	7-53 (2-16)	ND	7-53 (2-16)
	Christensen	9-18 (2-4)	9-18 (2-4)	4.5-18 (1-4)	9-72 (2-16)	0.8-1.6 (0.25-0.5)	0.8-1.6 (0.25-0.5)	0.8-1.6 (0.25-0.5)	0.8-1.6 (0.25-0.5)
541	RPMI	4.5 (1)	4.5-9 (1-2)	ND	4.5-9 (1-2)	7-53 (2-16)	13-53 (4-16)	ND	13-53 (4-16)
	Christensen	2.2-4.5 (0.5-1)	4.5 (1)	2.2-4.5 (0.5-1)	4.5-9 (1-2)	0.8-1.6 (0.25-0.5)	0.8-3 (0.25-1)	0.8-1.6 (0.25-0.5)	0.8-53 (0.25-16)
542	RPMI	9 (2)	9-18 (2-4)	ND	36-144 (8-32)	7-53 (2-16)	13-53 (4-16)	ND	7-53 (2-16)
	Christensen	≤1-4.5 (≤0.25-1)	2.2-4.5 (0.5-1)	≤1-4.5 (≤0.25-1)	18-36 (4-8)	0.4-1.6 (0.12-0.5)	0.4-1.6 (0.12-0.5)	0.4-1.6 (0.12-0.5)	0.8-53 (0.25-16)
543	RPMI	4.5 (1)	4.5-9 (1-2)	ND	4.5-9 (1-2)	3 (1)	3-7 (1-2)	ND	3-7 (1-2)
	Christensen	≤1-4.5 (≤0.25-1)	≤1-4.5 (≤0.25-1)	≤1-4.5 (≤0.25-1)	4.5-18 (1-4)	0.8-1.6 (0.25-0.5)	0.8-1.6 (0.25-0.5)	0.8-1.6 (0.25-0.5)	0.8-1.6 (0.25-0.5)
544A	RPMI	9 (2)	9 (2)	ND	9-18 (2-4)	7-13 (2-4)	7-26 (2-8)	ND	7-26 (2-8)
	Christensen	4.5-9 (1-2)	4.5-9 (1-2)	4.5-9 (1-2)	4.5-18 (1-4)	1.6 (0.5)	1.6 (0.5)	0.8-1.6 (0.25-0.5)	1.6 (0.5)
545A	RPMI	4.5-9 (1-2)	4.5-9 (1-2)	ND	4.5-9 (1-2)	7-53 (2-16)	7-53 (2-16)	ND	7-53 (2-16)
	Christensen	4.5 (1)	4.5 (1)	4.5 (1)	4.5-9 (1-2)	1.6-3 (0.5-1)	1.6-3 (0.5-1)	1.6 (0.5)	1.6 (0.5)
CAP59	RPMI	4.5 (1)	4.5-9 (1-2)	ND	4.5-9 (1-2)	3-7 (1-2)	3-7 (1-2)	ND	7 (2)
	Christensen	4.5 (1)	4.5-9 (1-2)	4.5-9 (1-2)	4.5-9 (1-2)	1.6 (0.5)	1.6 (0.5)	1.6 (0.5)	1.6 (0.5)

Table S3. Susceptibility of clinical isolates of *Cryptococcus neoformans* to novel urease inhibitors AF55 and AF57 in different culture media. Minimum Inhibitory Concentration values (IC_{50} and IC_{90}: 50% and 90% inhibition of fungal growth, respectively), Minimum Inhibitory Concentration of Urease Activity (IC_{URE}) and Minimum Fungicidal Concentration (MFC) were performed in RPMI and Christensen broth and are expressed in µM and µg/mL (in parenthesis). ND: not determined.

**Clinical isolates**	**Media**	**AF55**				**AF57**			
		IC_{50}	IC_{90}	IC_{URE}	MFC	IC_{50}	IC_{90}	IC_{URE}	MFC
H99	RPMI	6-12 (2-4)	6-12 (2-4)	ND	25 (8)	3 (1)	6 (2)	ND	6 (2)
	Christensen	3-6 (1-2)	3-6 (1-2)	3-6 (1-2)	3-6 (1-2)	0.3 (0.12)	0.3-0.6 (0.12-0.25)	0.3 (0.12)	0.3-0.6 (0.12-0.25)
541	RPMI	12 (4)	12-25 (4-8)	ND	25 (8)	3 (1)	3 (1)	ND	3 (1)
	Christensen	3-6 (1-2)	3-6 (1-2)	3-6 (1-2)	3-6 (1-2)	≤0.09-0.3 (≤0.03-0.12)	0.17-0.6 (0.06-0.25)	≤0.09 (≤0.03)	0.3-0.6 (0.12-0.25)
542	RPMI	12-25 (4-8)	25 (8)	ND	25 (8)	6 (2)	12 (4)	ND	47 (16)
	Christensen	6 (2)	6 (2)	1.5-6 (0.5-2)	6 (2)	0.3-1.2 (0.12-0.5)	0.6-1.2 (0.25-0.5)	0.6 (0.25)	0.6-1.2 (0.25-0.5)
543	RPMI	6-12 (2-4)	6-12 (2-4)	ND	12 (4)	3 (1)	3 (1)	ND	3 (1)
	Christensen	3-6 (1-2)	3-6 (1-2)	3-6 (1-2)	3-6 (1-2)	≤0.09-0.3 (≤0.03-0.12)	0.17-0.3 (0.06-0.12)	0.17 (0.06)	0.3 (0.12)
544A	RPMI	12 (4)	12 (4)	ND	12 (4)	3 (1)	3 (1)	ND	6 (2)
	Christensen	3-6 (1-2)	3-6 (1-2)	3-6 (1-2)	3-6 (1-2)	≤0.09-0.3 (≤0.03-0.12)	0.17-0.3 (0.06-0.12)	≤0.09-0.3 (≤0.03-0.12)	0.17-0.3 (0.06-0.12)
545A	RPMI	12 (4)	12 (4)	ND	12 (4)	3 (1)	3 (1)	ND	3 (1)
	Christensen	3-6 (1-2)	6 (2)	3-6 (1-2)	6 (2)	0.17-0.6 (0.06-0.25)	0.3-0.6 (0.12-0.25)	0.3-0.6 (0.12-0.25)	0.3-0.6 (0.12-0.25)
CAP59	RPMI	3-12 (1-4)	6-12 (2-4)	ND	12 (4)	3 (1)	3-6 (1-2)	ND	3-6 (1-2)
	Christensen	1.5-6 (0.5-2)	1.5-6 (0.5-2)	1.5-6 (0.5-2)	1.5-6 (0.5-2)	0.17-0.6 (0.06-0.25)	0.3-0.6 (0.12-0.25)	0.3-0.6 (0.12-0.25)	0.3-0.6 (0.12-0.25)

Table S4. Susceptibility of clinical isolates of *Cryptococcus gattii* to novel urease inhibitors AF19 and AF36 in different culture media. Minimum Inhibitory Concentration values (IC_{50} and IC_{90}: 50% and 90% inhibition of fungal growth, respectively), Minimum Inhibitory Concentration of Urease Activity (IC_{URE}) and Minimum Fungicidal Concentration (MFC) were performed in RPMI and Christensen broth and are expressed in µM and µg/mL (in parenthesis). ND: not determined.

Clinical isolates	Media	AF19				AF36			
		IC_{50}	IC_{90}	IC_{URE}	MFC	IC_{50}	IC_{90}	IC_{URE}	MFC
R265	RPMI	18-36<br>(4-8)	18-36<br>(4-8)	ND	18-143<br>(4-32)	7<br>(2)	7<br>(2)	ND	7<br>(2)
	Christensen	4.5<br>(1)	4.5<br>(1)	2.2-4.5<br>(0.5-1)	18-36<br>(4-8)	1.6<br>(0.5)	1.6<br>(0.5)	1.6<br>(0.5)	1.6<br>(0.5)
525	RPMI	9-18<br>(2-4)	18<br>(4)	ND	18-143<br>(4-32)	7<br>(2)	7<br>(2)	ND	7-14<br>(2-4)
	Christensen	≤1-4.5<br>(≤0.25-1)	≤1-4.5<br>(≤0.25-1)	≤1-4.5<br>(≤0.25-1)	9-18<br>(2-4)	0.4-0.8<br>(0.12-0.25)	0.4-0.8<br>(0.12-0.25)	0.2-0.4<br>(0.06-0.12)	0.8<br>(0.25)
527	RPMI	9<br>(2)	9<br>(2)	ND	9<br>(2)	7<br>(2)	7<br>(2)	ND	7<br>(2)
	Christensen	4.5<br>(1)	4.5<br>(1)	4.5<br>(1)	9<br>(2)	0.4-0.8<br>(0.12-0.25)	0.4-0.8<br>(0.12-0.25)	0.2-0.4<br>(0.06-0.12)	0.8<br>(0.25)
616A	RPMI	9-18<br>(2-4)	18<br>(4)	ND	36<br>(8)	1.6-3.2<br>(0.5-1)	1.6-3.2<br>(0.5-1)	ND	1.6-3.2<br>(0.5-1)
	Christensen	4.5<br>(1)	4.5<br>(1)	4.5<br>(1)	4.5<br>(1)	0.8-1.6<br>(0.25-0.5)	0.8-1.6<br>(0.25-0.5)	0.4-1.6<br>(0.12-0.5)	0.8-1.6<br>(0.25-0.5)
652A	RPMI	9<br>(2)	9<br>(2)	ND	18<br>(4)	7<br>(2)	7<br>(2)	ND	7<br>(2)
	Christensen	4.5<br>(1)	4.5<br>(1)	2.2<br>(0.5)	4.5<br>(1)	0.4<br>(0.12)	0.4<br>(0.12)	0.4<br>(0.12)	0.4<br>(0.12)
ATCC 56990	RPMI	9-18<br>(2-4)	9-18<br>(2-4)	ND	9-18<br>(2-4)	7<br>(2)	7-14<br>(2-4)	ND	7-14<br>(2-4)
	Christensen	4.5<br>(1)	4.5-9<br>(1-2)	4.5<br>(1)	4.5-9<br>(1-2)	0.4-0.8<br>(0.12-0.25)	0.4-0.8<br>(0.12-0.25)	0.4-0.8<br>(0.12-0.25)	0.8-1.6<br>(0.25-0.5)

Table S5. Susceptibility of clinical isolates of *Cryptococcus gattii* to novel urease inhibitors AF55 and AF57 in different culture media. Minimum Inhibitory Concentration values (IC_{50} and IC_{90}: 50% and 90% inhibition of fungal growth, respectively), Minimum Inhibitory Concentration of Urease Activity (IC_{URE}) and Minimum Fungicidal Concentration (MFC) were performed in RPMI and Christensen broth and are expressed in µM and µg/mL (in parenthesis). ND: not determined. ND*: no growth detected even in control.

**Clinical isolates**	**Media**	**AF55**				**AF57**			
		IC_{50}	IC_{90}	IC_{URE}	MFC	IC_{50}	IC_{90}	IC_{URE}	MFC
R265	RPMI	6-12 (2-4)	12 (4)	ND	12 (4)	3 (1)	3 (1)	ND	3 (1)
	Christensen	1.5-6 (0.5-2)	1.5-6 (0.5-2)	1.5-6 (0.5-2)	1.5-6 (0.5-2)	0.3 (0.12)	0.6 (0.25)	0.3 (0.12)	0.6 (0.25)
525	RPMI	6-12 (2-4)	6-12 (2-4)	ND	12 (4)	3 (1)	6 (2)	ND	6 (2)
	Christensen	1.5-3 (0.5-1)	1.5-3 (0.5-1)	0.7-3 (0.25-1)	1.5-3 (0.5-1)	0.1 (0.06)	0.3 (0.12)	0.1 (0.06)	0.3 (0.12)
527	RPMI	12 (4)	12 (4)	ND	12 (4)	3 (1)	6 (2)	ND	6 (2)
	Christensen	0.7-3 (0.25-1)	0.7-3 (0.25-1)	0.3-3 (0.12-1)	0.7-6 (0.25-2)	0.1 (0.06)	0.1 (0.06)	0.1 (0.06)	0.1 (0.06)
616A	RPMI	6 (2)	6-12 (2-4)	ND	12 (4)	0.3-6 (0.12-2)	0.3-6 (0.12-2)	ND	0.6-6 (0.25-2)
	Christensen	1.5-3 (0.5-1)	1.5-3 (0.5-1)	1.5-3 (0.5-1)	1.5-3 (0.5-1)	ND*	ND*	ND*	ND*
652A	RPMI	6-12 (2-4)	6-12 (2-4)	ND	12 (4)	0.3-6 (0.12-2)	0.3-6 (0.12-2)	ND	0.6-6 (0.25-2)
	Christensen	0.7-3 (0.25-1)	0.7-3 (0.25-1)	0.7-3 (0.25-1)	1.5-3 (0.5-1)	0.1 (0.06)	0.1 (0.06)	0.1 (0.06)	0.1 (0.06)
ATCC 56990	RPMI	6 (2)	6-12 (2-4)	ND	12 (4)	0.3-3 (0.12-1)	0.6-6 (0.25-2)	ND	0.6-6 (0.25-2)
	Christensen	1.5-3 (0.5-1)	1.5-3 (0.5-1)	1.5-3 (0.5-1)	1.5-3 (0.5-1)	0.1 (0.06)	0.3 (0.12)	0.1 (0.06)	0.3 (0.12)

Table S6. Susceptibility of clinical isolates of *Cryptococcus neoformans* to standard antifungals in different culture media. Minimum Inhibitory Concentration values (IC_{50} and IC_{90}: 50% and 90% inhibition of fungal growth, respectively), Minimum Inhibitory Concentration of Urease Activity (IC_{URE}) and Minimum Fungicidal Concentration (MFC) were performed in RPMI and Christensen broth and are expressed in μg/mL. ND: not determined.

Clinical isolates	Media	5-Flucytosine				Amphotericin B				Fluconazole			
		IC_{50}	IC_{90}	IC_{URE}	MFC	IC_{50}	IC_{90}	IC_{URE}	MFC	IC_{50}	IC_{90}	IC_{URE}	MFC
H99	RPMI	2-4	2-4	ND	16->16	0.06-0.12	0.06-0.12	ND	0.12-0.25	1-4	2-4	ND	16->16
	Christensen	1-4	2-8	2-4	16->16	0.06	0.06-0.12	0.12	0.12-0.25	1-4	2-4	2-4	16->16
541	RPMI	1	2	ND	8-16	0.12	0.12-0.25	ND	0.25	0.25-0.5	0.5-1	ND	1-2
	Christensen	2	4	2	>16	0.06-0.12	0.12	0.06-0.12	0.12-0.25	1	2	1	4
542	RPMI	4	4-8	ND	>16	0.06-0.25	0.12-0.25	ND	0.12-0.5	8-16	16->16	ND	>16
	Christensen	2	4	2	>16	≤0.03-0.06	≤0.03-0.12	≤0.03-0.12	≤0.03-0.12	8	16	1	>16
543	RPMI	1	2	ND	8->16	0.06	0.06-0.12	ND	0.12	0.25-1	0.5-2	ND	1-2
	Christensen	2	4	2	16->16	≤0.03	≤0.03-0.06	≤0.03-0.06	≤0.03-0.12	1	2	2	4
544A	RPMI	4	8	ND	8-16	0.12-0.25	0.25	ND	0.25	1-4	4-8	ND	8
	Christensen	4-8	8-16	4-8	16->16	0.12-0.25	0.12-0.25	0.12-0.25	0.12-0.25	2	4	4	8
545A	RPMI	4	4-8	ND	16	0.12	0.12	ND	0.12-0.5	2-4	4-8	ND	4-16
	Christensen	4	4-8	2-4	16	0.06-0.12	0.12	0.06-0.12	0.12-0.25	2	4	4	4
CAP59	RPMI	2	4	ND	4	≤0.03-0.06	≤0.03-0.06	ND	≤0.03-0.12	2	4	ND	4
	Christensen	2	4	2	4-8	≤0.03	≤0.03	≤0.03	≤0.03-0.06	2	4	4	4

ND: not determined.

Table S7. Susceptibility of clinical isolates of *Cryptococcus gattii* to standard antifungals in different culture media. Minimum Inhibitory Concentration values (IC_{50} and IC_{90}: 50% and 90% inhibition of fungal growth, respectively), Minimum Inhibitory Concentration of Urease Activity (IC_{URE}) and Minimum Fungicidal Concentration (MFC) were performed in RPMI and Christensen broth and are expressed in µg/mL.

**Clinical isolates**	**Media**	**5-Flucytosine**				**Amphotericin B**				**Fluconazole**			
		IC_{50}	IC_{90}	IC_{URE}	MFC	IC_{50}	IC_{90}	IC_{URE}	MFC	IC_{50}	IC_{90}	IC_{URE}	MFC
R265	RPMI	4	4	ND	>16	0.06	0.12	ND	0.12	16	>16	ND	>16
	Christensen	2	4	2	>16	0,03	0.06	0.06	0.06	8	16	16	>16
525	RPMI	2	4	ND	16->16	0.12-0.25	0.12-0.25	ND	0.12-0.25	8-16	16->16	ND	>16
	Christensen	1	2	2	16	≤0.03-0.06	≤0.03-0.06	≤0.03-0.06	0.06	8	16	4	>16
527	RPMI	4	4	ND	>16	0.06-0.12	0.06-0.12	ND	0.06-0.12	4-16	8->16	ND	>16
	Christensen	4	4	1-4	>16	≤0.03	≤0.03	≤0.03	≤0.03	8	16	16	>16
616A	RPMI	2	2	ND	>16	0.12	0.12-0.25	ND	0.25	4-8	8-16	ND	>16
	Christensen	0.5	0.5	0.5	16	0.06	0.06	0.06	0.25	2	4	4	>16
652A	RPMI	4	4-8	ND	16->16	0.12-0.25	0.12-0.25	ND	0.25	4-8	8-16	ND	>16
	Christensen	4-8	8	8	16	0,03-0.06	0.06	0.03-0.06	0.06-0.12	4	8	8	>16
ATCC 56990	RPMI	2	2-4	ND	4	0.12-0.25	0.12-0.25	ND	0.25	4-8	8-16	ND	16->16
	Christensen	1	1-2	1-2	16	0.06	0.06	0.03	0.25	4	8	8	16

ND: not determined.

Table S8. Checkerboard assay of combinations of urease inhibitors (AF19 and AF36) with the standard antifungals 5-flucytosine (5-FC), amphotericin B (AMB) and fluconazole (FLC) on *Cryptococcus neoformans* H99 cells. MIC: minimum inhibitory concentration. The result (R) is based on the fractional inhibitory concentration index (FICI) when ≤ 0.5 = synergism (S); FICI > 0.5 and < 4 = indifferent (I); FICI ≥ 4 = antagonism (A).

Cryptococcus neoformans **H99**							
MIC (μg/mL)		FICI	R	MIC (μg/mL)		FICI	R
AF19	FLC			AF36	FLC		
0	4	1	I	1	0	0.5	I
0.25	4	1.12	I	1	0.5	0.62	I
0.5	2	0.75	I	1	1	0.75	I
1	2	1	I	1	2	1	I
2	2	1.5	I	1	4	1.5	I
2	1	1.25	I	0.5	8	2.25	I
2	0.5	1.12	I	0.25	8	2.12	I
2	0	1	I	0	8	2	I
AF19	AMB	FICI	R	AF36	AMB	FICI	R
0	0.06	1	I	2	0	1	I
0.25	0.06	1.12	I	2	0.0075	1.06	I
2	0.03	1.5	I	2	0.015	1.25	I
2	0.015	1.25	I	2	0.03	1.25	I
2	0.0075	1.12	I	2	0.06	1.5	I
2	0	1	I	1	0.06	1	I
AF19	5-FC	FICI	R	0.5	0.12	1.25	I
0	4	1	I	0.25	0.12	1.12	I
0.25	2	0.62	I	0	0.12	1	I
0.5	2	0.75	I	AF36	5-FC	FICI	R
1	0.5	0.62	I	1	0	0.5	I
2	0	1	I	1	0.5	0.62	I
				1	1	0.75	I
				1	2	1	I
				0.5	4	1.25	I
				0.25	8	2.12	I
				0	8	2	I

Table S9. Checkerboard assay of combinations of urease inhibitors (AF55 and AF57) with the standard antifungals 5-flucytosine (5-FC), amphotericin B (AMB) and fluconazole (FLC) on *Cryptococcus neoformans* H99 cells. MIC: minimum inhibitory concentration. The result (R) is based on the fractional inhibitory concentration index (FICI) when ≤ 0.5 = synergism (S); FICI > 0.5 and < 4 = indifferent (I); FICI ≥ 4 = antagonism (A).

Cryptococcus neoformans H99

MIC (µg/mL)		FICI	R
AF55	FLC		
2	0	0.5	I
2	0.5	0.62	I
2	1	0.75	I
2	2	1	I
1	4	1.25	I
0.5	4	1.12	I
0	4	1	I

AF55	AMB	FICI	R
4	0	1	I
4	0.0075	1.12	I
4	0.015	1.25	I
4	0.03	1.5	I
2	0.06	1.5	I
1	0.06	1.25	I
0.5	0.06	1.12	I
0	0.06	1	I

AF55	5-FC	FICI	R
2	0	0.5	I
2	0.5	0.62	I
2	1	0.75	I
2	2	1	I
1	4	1.25	I
0.5	4	1.12	I
0	4	1	I

MIC (µg/mL)		FICI	R
AF57	FLC		
2	0	1	I
2	0.5	1.12	I
2	1	1.25	I
2	2	1.5	I
1	2	1	I
0.5	2	0.75	I
0.25	2	0.62	I
0	2	0.5	I

AF57	AMB	FICI	R
2	0	1	I
2	0.0075	1.06	I
2	0.015	1.25	I
2	0.03	1.25	I
2	0.06	1.5	I
2	0.12	2	I
1	0.12	2	I
0.5	0.12	2	I
0.25	0.12	2	I
0	0.12	2	I

AF57	5-FC	FICI	R
4	0	2	I
2	0.5	1.12	I
2	1	1.25	I
2	2	1.5	I
1	2	1	I
0.5	2	0.75	I
0.25	2	0.62	I
0	2	0.5	I

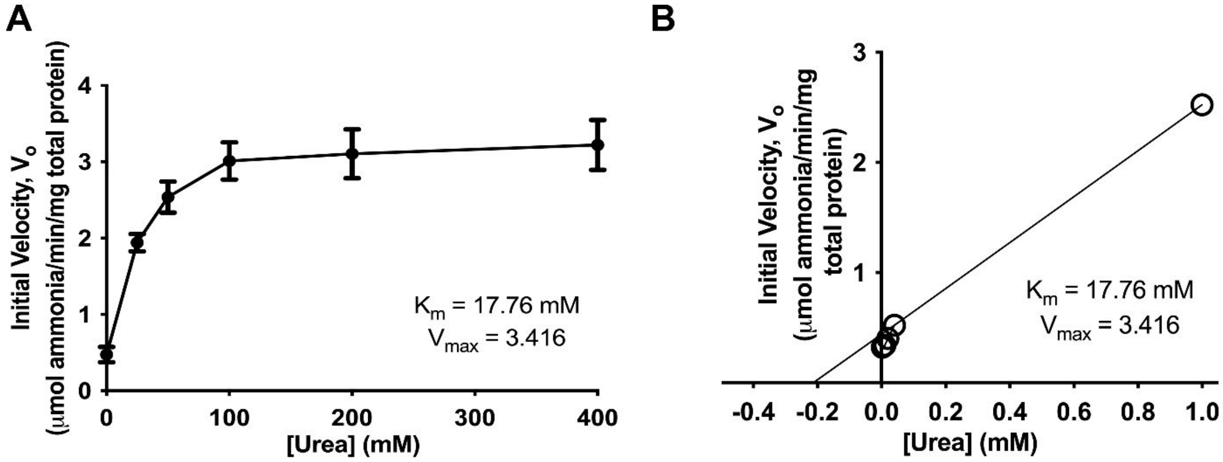

Figure S1. ***Cryptococcus neoformans*** **H99 urease kinetics plots of Michaelis-Menten (A) and Lineweaver-Burk (B).** The urease activity in crude protein extract (150-200 μg) was measured for 25, 50, 100, 200, and 400 mM urea and calculated using the ammonia production (μmol ammonia/min/mg total protein). Each data point represents the mean with standard error of four biological replicates, each one performed in triplicate.

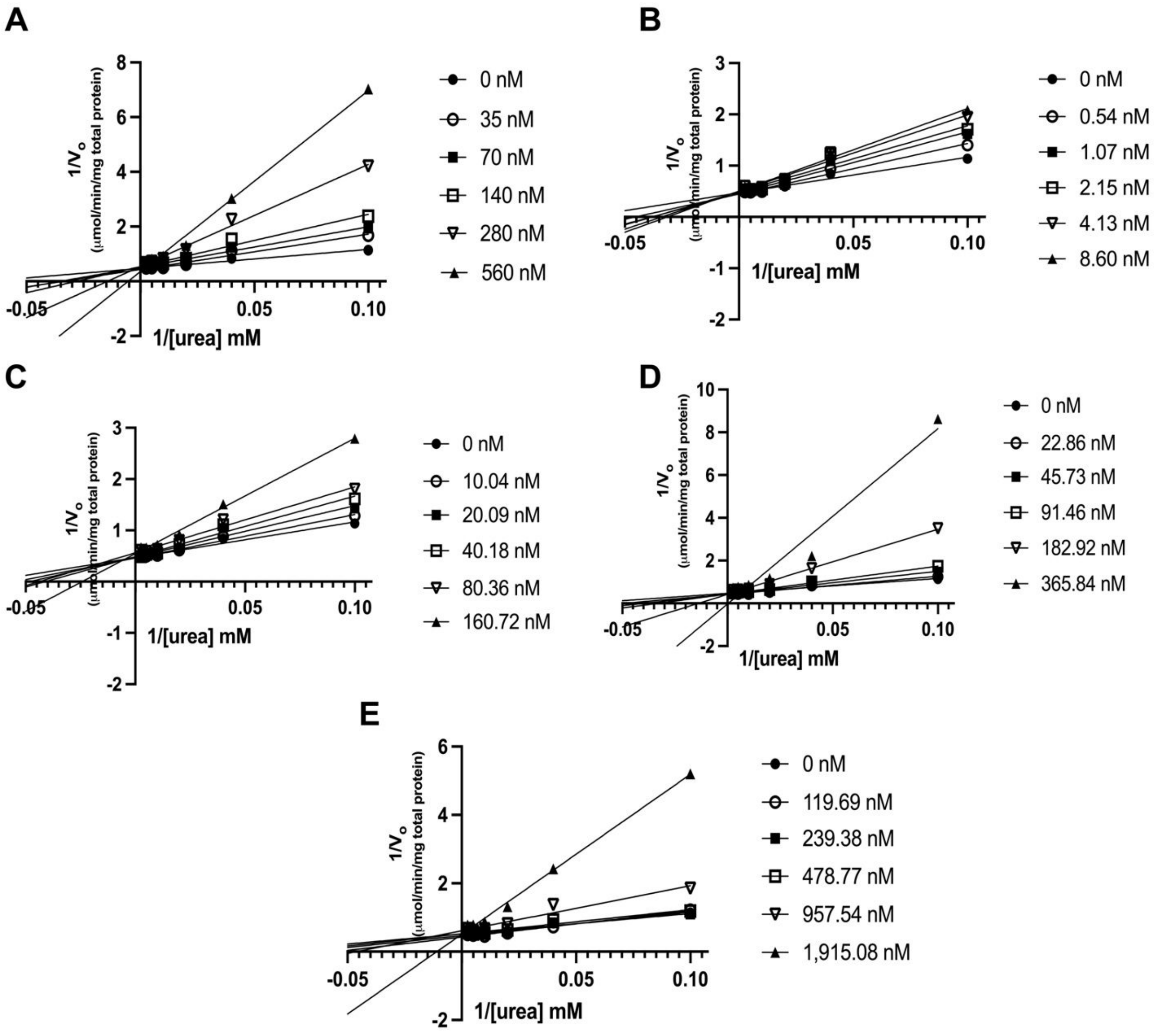

Figure S2. Lineweaver-Burk double reciprocal linear fitting of *Cryptococcus neoformans* urease from crude extract inhibited by (A) AF19, (B) AF36, (C) AF55, (D) AF57, and (E) acetohydroxamic acid (AHA). The urease activity in crude protein extract (150-200 µg) was measured for 10, 25, 50, 100, 200, and 400 mM urea and calculated based on the ammonia production (µmol ammonia/min/mg total protein). Each data point represents the mean of four separated experiments, each one performed in triplicate.

Table S10. Kinetic parameters of *Cryptococcus neoformans* H99 urease obtained from crude protein extract and inhibited by novel synthetic urease inhibitors (UREi) and the standard UREi acetohydroxamic acid (AHA).

UREi	Inhibition type	[I] (nM)	K_m or $_{app}K_m$ (mM)	V_{max} or $_{app}V_{max}$ (µmol ammonia/min/mg total protein)	K_i (nM)
AHA	Non-competitive	0	17.82	2.244	-
		119.69	14.42	2.242	2,312
		239.38	10.47	1.936	
		478.77	14.21	1.972	
		957.54	21.64	1.709	
		1,915.08	50.02	1.551	
AF19	Competitive	0	17.82	2.244	-
		35	30.03	2.405	66.88
		70	33.24	2.086	
		140	38.02	1.996	
		280	55.04	1.755	
		560	65.84	1.665	
AF36	Non-competitive	0	17.82	2.244	-
		0.54	24.92	2.359	32.32
		1.07	25.60	2.235	
		2.15	28.85	2.197	
		4.30	28.86	2.040	
		8.60	28.65	1.968	
AF55	Mixed	0	17.82	2.244	-
		10.04	21.53	2.358	149.1
		20.09	25.65	2.355	
		40.18	31.19	2.356	
		80.36	25.42	1.963	
		160.72	32.02	1.848	
AF57	Competitive	0	17.82	2.244	-
		22.86	17.58	2.379	64.30
		45.73	21.05	2.329	
		91.46	20.98	2.212	
		182.92	27.72	1.841	
		365.84	42.79	1.574	

Concentration of inhibitors ([I]) and inhibition constant (K_i) are expressed in nM. Michaelis-Menten constants for free enzyme (K_m and V_{max}) or inhibited (appK_m and appV_{max}) are expressed in mM and µmol ammonia/min/mg total protein, respectively. The values were calculated using GraphPad Prism 10.0 and represents the mean with standard error of three biological replicates, each one performed in duplicate.

Table S11. Canonical SMILES representations of the novel urease inhibitors.

Urease inhibitors	Canonical SMILES
AF19	OC1=CC=CC=C1\N=C\C=C\C1=CC=CC=C1
AF36	O=C(NC(=S)NC1=CC=CC=C1)C1=CC=CC=C1
AF55	CCOC(=O)C1=C(C)NC(=[Se])NC1C1=CC=CC=C1
AF56	CCOC(=O)C1=C(C)NC(=[Se])NC1C1=C(O)C=CC=C1
AF57	CCOC(=O)C1=C(C)NC(=[Se])NC1C1=CC(O)=CC=C1
AF58	CCOC(=O)C1=C(C)NC(=[Se])NC1C1=CC=C(O)C=C1

Table S12. Toxicological properties of novel urease inhibitors using Osiris property explorer.

Toxicological properties	AF19	AF36	AF55	AF56	AF57	AF58
Mutagenic (Yes/No)	Yes	No	No	No	No	No
Tumorigenic (Yes/No)	No	No	No	No	No	No
Irritant (Yes/No)	No	No	No	No	No	No
Reproductive effect (Yes/No)	No	No	No	No	No	No

Table S13. ***In silico*** **pharmacokinetic and toxicity parameters of novel urease inhibitors predicted by SwissADME and pkCSM.**

Property	Model Name	Urease inhibitors			
		AF19	AF36	AF55	AF58
Absorption	Skin permeability (log Kp)	-1.95	-2.75	-3.40	-3.72
	P-glycoprotein substrate (Yes/No)	No	Yes	Yes	Yes
	P-glycoprotein I inhibitor (Yes/No)	No	No	No	No
	P-glycoprotein II inhibitor (Yes/No)	No	No	No	No
Distribution	Fraction unbound (human) (Fu)	0.00	0.15	0.33	0.40
Metabolism	CYP2D6 substrate (Yes/No)	No	Yes	No	No
	CYP3A4 substrate (Yes/No)	Yes	Yes	No	No
	CYP2D6 inhibitor (Yes/No)	No	No	No	No
	CYP3A4 inhibitor (Yes/No)	No	No	No	No
Toxicity	AMES toxicity (Yes/No)	Yes	No	No	No
